# AKI as a systemic syndrome and its impact on other organ systems

**DOI:** 10.1186/s13054-026-06252-x

**Published:** 2026-08-22

**Authors:** Benedetta Manca, Lui Forni, Hendrik Booke, Sven Meuth, Jay Koyner, Ashley La, Alexander Zarbock

**Affiliations:** 1 Department of Anaesthesiology, Intensive Care and Pain Medicine, University Hospital Münster, Albert-Schweitzer-Campus 1, building A1, 48149 Münster, Germany; 2 Department of Basic Biotechnological Sciences, Intensive Care and Perioperative Clinical Sciences, Catholic University of the Sacred Heart, Rome, Italy; 3 School of Medicine, University of Surrey, Guildford, UK; 4 Department of Intensive Care Medicine, Royal Surrey County Hospital, Egerton Road, Guildford, Surrey, GU2 7XX UK; 5 Section of Nephrology, Department of Medicine, University of Chicago, Chicago, IL USA

**Keywords:** Acute kidney injury, Distant organ injury, Inflammation, Cross-talk

## Abstract

Acute kidney injury (AKI) affects approximately 15% of all hospitalized individuals and nearly half of those admitted to intensive care units and is increasingly recognized as a systemic syndrome whose consequences extend well beyond structural and functional changes confined to the kidney. Experimental studies highlight the role of inflammation and accumulation of metabolites as pivotal drivers in the cross-talk between the kidneys and distant organ systems during AKI. Whereas clinical data reveals an association between AKI and non-renal organ dysfunctions and complications. This review outlines the pathophysiological mechanisms underlying AKI-associated remote organ dysfunction, focusing on five key organ systems: the lungs, the brain, the heart, the liver and the immune system. We discuss the direct consequences of reduced kidney function alongside the repercussions of the inflammatory cascade triggered by kidney cell injury and the resulting clinical implications. Recognizing that multi-organ failure significantly influences patients’ morbidity and mortality, identifying specific pathways of organ cross-talk is essential to optimize clinical management. Finally, we evaluate novel endpoints for future AKI trials. Given that traditional long-term metrics, such as Major Adverse Kidney Events (MAKE), present significant design challenges in non-enriched populations, we analyze alternative and surrogate outcomes, including those directly related to the remote organ complications of AKI.

## Background

Acute kidney injury (AKI) is now recognized as a major health problem affecting millions of patients globally. General estimates of the AKI incidence in the past varied, with data from different populations ranging from 114 to 174 people per 10,000 person-years, or approximately 13.3 million worldwide in 2017 [1, 2]. Reported AKI incidence is highest in low- and middle-income countries with AKI affecting approximately 15% of all hospitalized patients and about half of critically ill patients in the highest income areas [1, 2]. AKI is associated with not only increased mortality but also increased morbidity [3–5]. Patients who survive an episode of AKI have an increased risk of developing chronic kidney disease (CKD) [6–10]. Outcomes in AKI are affected not only by the original cause of the AKI, but also by the duration and severity of kidney dysfunction as well as the baseline status of the patient, including the presence of CKD.

Although kidney replacement therapy (KRT) is available, the cause of the high mortality rate in patients with severe AKI is still not fully elucidated. AKI is not an isolated organ failure of the kidney; rather, due to the kidney’s numerous functions, other organs are also affected. Injury of distant organs is considered an important cause of complication and mortality, given that multi-organ dysfunction is often observed in AKI patients and the number of organ dysfunctions is associated with increased mortality [11, 12]. Patients with a dialysis-dependent AKI have a considerably higher mortality rate compared to patients with end-stage kidney disease (ESKD) [13], suggesting that, in addition to impaired kidney function in AKI, other factors contribute to the poor prognosis. These data suggest that organ dysfunction induced by AKI plays a significant role in the prognosis of critically ill patients. Experimental studies have uncovered various mechanisms contributing to AKI-induced distant organ injury. Mechanisms involving the immune system, lungs, heart, liver, brain, and gut have been demonstrated. These mechanisms are organ-specific and diverse, encompassing immune cell responses, oxidative stress, and inflammatory cytokines. These organ interactions, established in animal studies, have been corroborated in clinical observational studies [12, 13].

This review article briefly outlines the molecular mechanisms of AKI-associated remote organ dysfunction, the epidemiology of complications, and the clinical outcomes reported in the literature. The final section discusses novel endpoints for AKI trials, taking into account organ interactions during AKI and the resulting complications.

## Consequences of AKI

AKI is associated with an increase in hospital length of stay (LOS), ICU-LOS, and higher morbidity and short and long-mortality [2, 14]. The 2012 Kidney Disease Improving Global Outcomes (KDIGO) guidelines provided a classification of AKI in three different stages of severity based on serum creatinine levels and hourly urine output (Table 1) [15]. A correlation with AKI stages can be observed, where KDIGO stage 1 shows a twofold increase in mortality whereas severe AKI (stage 2–3) shows an almost 7-fold increase in mortality [5]. Clinical presentation of AKI is highly heterogeneous and depends on the severity of the injury. Many symptoms can be explained by an acute loss of glomerular filtration capability, which leads to azotemia and accumulation of protons, electrolytes, toxins, and drugs. The accumulation of uremic toxins has a direct impact on other organ systems and can start a vicious cycle of nephrotoxicity leading to reduced glomerular filtration rate (GFR) with further accumulation of toxins and subsequent increased toxicity [16, 17]. The development of metabolic acidosis is associated with a particularly poor prognosis [18]. Uremic toxicity can lead to arrhythmias (e.g. due to hyperkalemia), tiredness (e.g. due to azotemia), and in some cases it can cause confusion or even coma (e.g. accumulation of toxins, drugs). Furthermore, reduced urine output can lead to fluid overload and congestion which can cause or worsen ventilatory problems and lead to venous congestion with direct impact on other organs such as the liver and the kidney itself. After the initial resuscitation phase reduced kidney function can also complicate fluid deresuscitation [19].

**Fig. 1:**
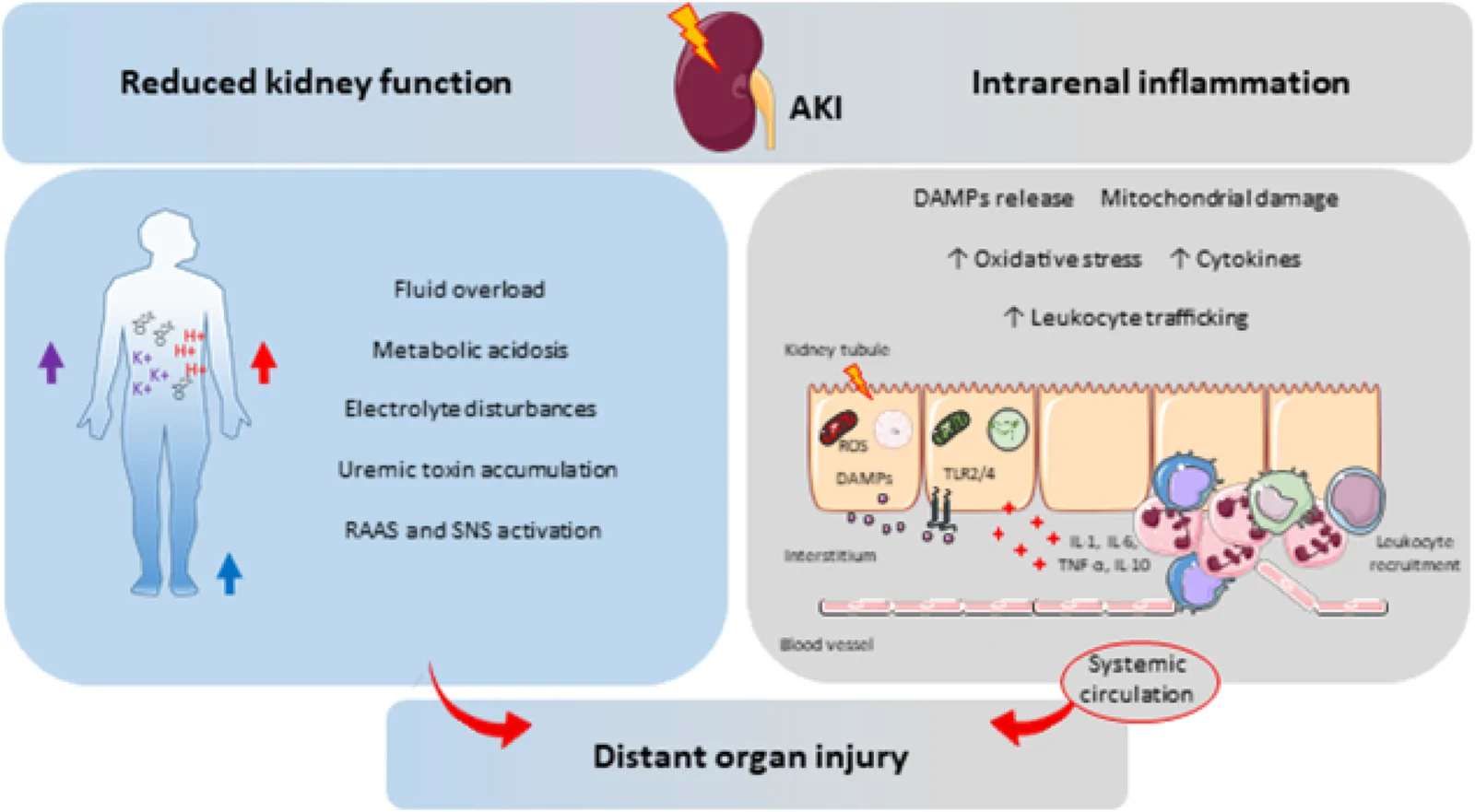
Mechanisms of AKI-induced distant organ injuries. AKI=kidney injury; DAMPs= damage associated molecular patterns; IL-1 = interleukin-1; IL-6 = interleukin-6; IL-10 = interleukin-10; RAAS = renin-angiotensin-aldosterone system; ROS = reactive oxygen species; SNS= sympathetic nervous system; TLR2/4 = toll-like receptor 2/4; TNF-α = tumor necrosis factor-alpha

Additionally, an episode of AKI may have longer-lasting effects on kidney function and increase the risk for the development or progression of CKD [20–23]. Even with apparent full recovery of kidney function the risk for ESKD stays elevated after single episodes of AKI [24–26]. The mechanisms underlying this progression are not solely explained by the loss of functioning nephrons but also by maladaptive repair mechanisms, disordered regeneration, and a vicious cycle of endothelial injury leading to hypoxia/ischemia and further endothelial injury [27–29]. As a result, AKI is regarded as a syndrome with multiple different consequences and not all clinical presentations of AKI can solely be explained by functional reductions of GFR [30]. Kidney cell injury and intrarenal inflammation play an important role in the pathophysiology of organ cross-talk in AKI and consequences thereof [31, 32]. AKI leads to (sterile) activation of kidney cells and local immune cells, including resident macrophages and dendritic cells, which causes the release of proinflammatory cytokines and subsequent systemic inflammation [32]. Over the past decades, the role of different immune cells (e.g. dendritic cells, macrophages), cytokines (e.g. interleukin-17 - IL-17, and tumor necrosis factor-alpha - TNF-α), and pattern recognition receptors (e.g. toll-like receptors - TLR) in development and progression of AKI was discovered. Moreover, epigenetic changes of fibroblasts, myofibroblasts, pericytes, and epithelial cells also play important roles in the progression of AKI and its consequences [33–37]. For example, after apparent recovery of kidney function profibrotic signaling might still persist locally [35, 38] and distantly through humoral pathways leading to long-term consequences in other organs [39]. This highlights AKI as a systemic syndrome with consequences going far beyond the acute complications resulting from acutely reduced GFR (Fig. 1).

The following section will address the impact of AKI on the lung, the brain, the heart, the liver and the immune system. For each organ system, the pathophysiological mechanisms and preclinical findings, focusing on inflammation triggered by AKI, will be discussed alongside clinical evidence, patient outcomes, and therapeutic strategies. Organ-specific damage biomarkers relevant to each of these systems, both experimental and clinically implemented, are summarized in Table 2 to complement the discussion.

**Table 1:** AKI severity stage according to KDIGO 2012 guidelines [15]

Stage	Serum creatinine	Urine output
1	1.5–1.9 times baseline OR ≥ 0.3 mg/dl (≥ 26.5mmol/l) increase	< 0.5 ml/kg/h for 6–12 h
2	2.0–2.9 times baseline	< 0.5 ml/kg/h for ≥ 12 h
3	3.0 times baseline OR Increase in serum creatinine to ≥ 4.0 mg/dl (≥ 353.6mmol/l) OR Initiation of renal replacement therapy OR, In patients < 18 years, decrease in eGFR to < 35 ml/min per 1.73 m2	< 0.3 ml/kg/h for ≥ 24 h OR Anuria for ≥ 12 h

eGFR = estimated glomerular filtration rate

**Table 2:** Biomarkers of organ injury

Organ/system	Biomarkers	Status	Purpose
Lung [40]	RAGE, SP-D, Ang-2	Experimental	Epithelial/endothelial injury; ALI
Brain [41, 42]	NfL, GFAP, UCH-L1, Tau S100B, NSE	Experimental Implemented	Brain dysfunction; prognosis Prognostication in TBI and cardiac arrest; still under evaluation for neuroprognostication in the critically ill
Heart [43]	BNP, cTn sST2, Galectin-3	Implemented Experimental	Diagnosis of HF/ACS Prognosis
Liver [44, 45]	Bilirubin, GGT, ALP AST, ALT miR-122, GLDH, K18/ccK18, HMGB-1	Implemented Implemented Experimental	Congestion Hepatocellular injury Hepatocellular injury
Infections [46]	PCT, CRP	Implemented	Infection and sepsis
Immune system [46]	IL-6, IL-8, IL-10	Experimental	Inflammation/immunoparalysis
Kidney [47]	SCr, UO, Cystatin C NGAL, TIMP-2·IGFBP7 CCL14, DKK3, proenkephalin A, KIM-1, L-FABP	Implemented Implemented Experimental	AKI diagnosis and staging, GFR estimation Not routinely. Early tubular injury/stress; risk stratification Persistent AKI, risk prediction, sub-phenotyping

ACS = acute coronary syndrome; AKI = acute kidney injury; ALI = acute lung injury; ALP = alkaline phosphatase; ALT = alanine aminotransferase; Ang-2 = angiopoietin-2; AST = aspartate aminotransferase; BNP = B-type natriuretic peptide; ccK18 = caspase-cleaved cytokeratin 18; CCL14 = C-C motif chemokine ligand 14; CRP = C-reactive protein; cTn = cardiac troponin; DKK3 = Dickkopf-3; GFAP = glial fibrillary acidic protein; GFR = glomerular filtration rate; GGT = gamma-glutamyl transferase; GLDH = glutamate dehydrogenase; HF = heart failure; HMGB-1 = high mobility group box 1; IGFBP7 = insulin-like growth factor-binding protein 7; IL = interleukin; K18 = cytokeratin 18; KIM-1 = kidney injury molecule-1; L-FABP = liver-type fatty acid-binding protein; miR-122 = microRNA-122; NfL = neurofilament light chain; NGAL = neutrophil gelatinase-associated lipocalin; NSE = neuron-specific enolase; PCT = procalcitonin; RAGE = receptor for advanced glycation end-products (soluble form, sRAGE); S100B = S100 calcium-binding protein B; SCr = serum creatinine; SP-D = surfactant protein D; sST2 = soluble suppression of tumorigenicity 2; Tau = tau protein; TBI = traumatic brain injury; TIMP-2 = tissue inhibitor of metalloproteinases-2; UCH-L1 = ubiquitin C-terminal hydrolase L1; UO = urine output

## AKI and its impact on organ systems

### Lung

**Fig. 2:**
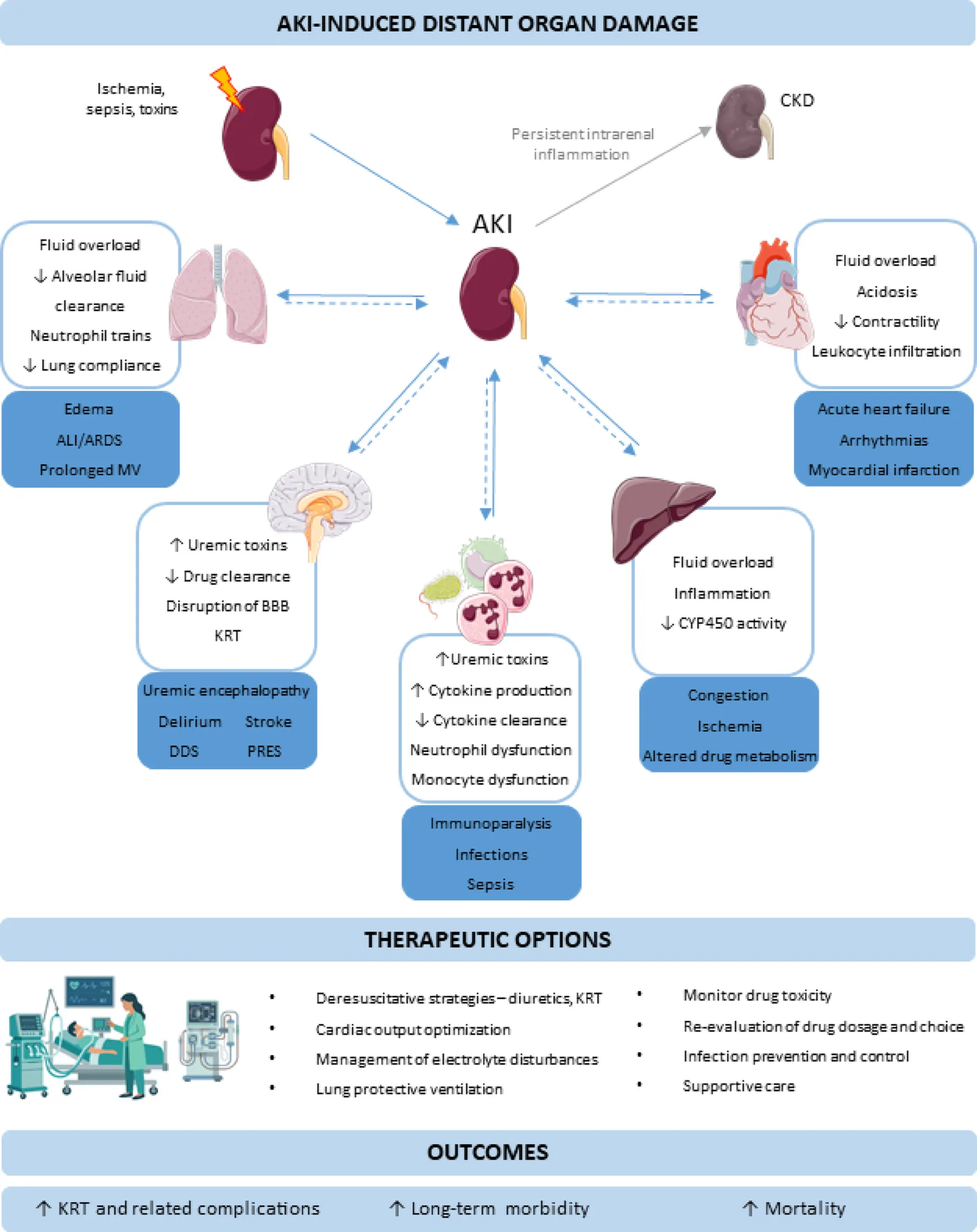
Bidirectional cross-talk between the kidney and the distant organ systems. Key cross-talk mechanisms, clinical manifestations of AKI-induced injury, therapeutic strategies and patient outcomes. AKI = acute kidney injury; ALI = acute lung injury; ARDS = acute respiratory distress syndrome; BBB = blood-brain barrier; CKD = chronic kidney disease; CYP450 = cytochrome P450; DDS = dialysis disequilibrium syndrome; KRT = kidney replacement therapy; MV = mechanical ventilation; PRES = posterior reversible encephalopathy syndrome

During the course of AKI, several mechanisms—most notably fluid overload—may contribute to the development of acute lung injury (ALI) [48]. In a small single-center study of patients with severe dialysis-requiring AKI, 47% developed pulmonary complications, mainly presenting as acute respiratory distress syndrome (ARDS) [49]. Consistent with this observation, 70%–85% of patients with AKI admitted to the ICU required mechanical ventilation across multiple studies, reflecting the high burden of respiratory failure in this population [50]. Conversely, in patients with ALI requiring mechanical ventilation, impaired venous return, systemic venous congestion, and low preload-dependent cardiac output (CO) may reduce renal perfusion pressure, thereby decreasing GFR [51]. This generates a vicious cycle that poses a significant threat to patients, clinicians and healthcare systems (Fig. 2). Furthermore, both organs are highly susceptible to systemic inflammation, which acts as a key driver of pathological crosstalk [48].

#### Consequences of AKI

During AKI, impaired sodium and water excretion can lead to life-threatening fluid overload [52]. In this setting, increased intravascular volume raises hydrostatic pressure, possibly leading to ventricular dysfunction and cardiogenic pulmonary edema [53–55]. This process is rapidly exacerbated in patients with pre-existing heart failure. Edema alters cell-to-cell interactions within the lung and disrupts tissue architecture to varying degrees, resulting in ALI and the need for respiratory support [56]. Accordingly, patients requiring mechanical ventilation who develop AKI exhibit higher plateau and driving pressures, reduced respiratory system compliance, and lower PaO₂/FiO₂ ratios, indicating more severe impairment of pulmonary function [57, 58].

Beyond fluid overload and metabolic disturbances AKI may also trigger intrarenal inflammation and the resulting inflammatory mediator release may independently induce distant organ damage. Preclinical models of AKI, including ischemia-reperfusion injury, have elucidated the mechanisms underlying the pathological crosstalk between kidneys and other organs [59]. In preclinical models of AKI-induced ALI, it has been demonstrated that inflammation contributes to pulmonary edema by increasing pulmonary capillary leak [60]. Injured tubular cells may release Damage-Associated Molecular Patterns (DAMPs)—including mitochondrial DNA, HMGB-1, and histones—which activate TLR4 signaling on tubular epithelial cells, with systemic release of proinflammatory cytokines [60–62]. These mediators increase pulmonary endothelial permeability, recruit leukocytes into the pulmonary tissue, and promote the development of non-cardiogenic pulmonary edema [54, 60]. A recent study showed that, in preclinical models of AKI-induced ALI, neutrophil recruitment to the lungs was predominantly confined to the alveolar capillaries, where neutrophils formed characteristic “neutrophil trains”, differently from direct pulmonary injury, where neutrophils primarily accumulated within the alveolar space [63]. These findings suggest that pulmonary injury in AKI is not solely a consequence of fluid overload, hemodynamic instability, or metabolic disturbances, but also involves distinct inflammatory pathways that contribute to lung dysfunction and disease progression. Furthermore, AKI may impair edema resolution via downregulation of alveolar epithelial sodium channels, Na⁺/K⁺-ATPase, and aquaporin-5, which participate in alveolar fluid clearance [64]. From a pathophysiological perspective, these alterations may justify the lack of response to fluid removal in some patients with refractory pulmonary edema [50, 65].

#### Clinical implications

Pulmonary complications in the context of AKI represent a major determinant of prognosis [50, 66]. A recent large retrospective single-center cohort study demonstrated that the coexistence of AKI and ARDS is associated with substantially worse short-term outcomes than either condition alone. ICU mortality reached 21%, compared with 4%–9% in patients with isolated AKI or ARDS, and was accompanied by longer ICU stays, prolonged mechanical ventilation, and increased use of KRT [66]. Mortality was highest when ARDS developed after AKI compared to the reverse (29% vs. 14%), suggesting that the temporal sequence of organ injury may influence prognosis. However, the retrospective observational design precludes causal inference, and the long-term consequences of AKI-associated lung injury remain poorly defined.

Given its independent association with increasing mortality, addressing fluid overload appears of paramount importance [56]. Whenever feasible, personalized fluid stewardship, deresuscitative strategies following initial stabilization, or ultrafiltration should be adopted to achieve a neutral or even negative fluid balance [19, 67]. In parallel, lung-protective mechanical ventilation remains essential, not only to limit pulmonary injury, but also because it may reduce the risk of AKI development or progression [58].

### Brain

The clinical association between AKI and cerebral dysfunction is consistently reported [68–71]. In the acute setting, uremic encephalopathy appears to affect up to 20% of patients with AKI admitted to the ICU [72], while a recent meta-analysis involving 158,694 patients reported a pooled delirium prevalence of 32%, increasing in parallel with AKI severity [73]. Conversely, the central nervous system has been reported to influence the course of AKI (Fig. 2), either through direct pathways or via immune system modulation [74].

#### Consequences of AKI

AKI might mediate acute brain injury through different mechanisms. The role of uremic toxins remains a subject of intense scrutiny. Urea and its metabolites accumulate due to both AKI and the administration of high protein nutrition in critically ill patients [75]. Preclinical models suggest that a uremic milieu may act as a potent stimulus for renal recovery [76]. Nonetheless, specific urea-cycle metabolites, such as guanidino compounds and protein-bound solutes, are known to alter synaptic transmission, induce direct cellular injury, and promote neuroinflammation [70, 77]. Toxic effects likely emerge only in the setting of elevated urea levels. In a secondary analysis of the AKIKI 2 trial, patients managed according to a ‘more-delayed’ KRT strategy (BUN > 140 mg/dL) spent more days in coma compared to those in the ‘delayed’ strategy group (BUN > 112 mg/dL), suggesting that prolonged exposure to higher levels of uremic toxins may exacerbate cerebral dysfunction [78]. Furthermore, rapid urea clearance, particularly during intermittent KRT, sharply reduces extracellular tonicity, creating an osmotic gradient that might cause cerebral edema and, clinically, a broad spectrum of neurological symptoms known as dialysis disequilibrium syndrome [79]. Altered mental status, headache, seizures, and visual disturbances during AKI may also be related to posterior reversible encephalopathy syndrome (PRES) [80]. Driven by fluid retention and renin-angiotensin-aldosterone system (RAAS) activation, AKI-induced hypertension can trigger PRES via impaired cerebral autoregulation. However, vascular endothelial injury and blood-brain barrier (BBB) disruption also cause parieto-occipital vasogenic edema independently of severe hypertension [80]. In this clinical scenario, MRI might be essential for differential diagnosis [80].

Furthermore, metabolic acidosis secondary to AKI can directly impair neuronal function. Activation of acid-sensing ion channels on neuronal membranes triggers an influx of calcium and sodium ions, leading to depolarization, neuronal injury, and potentially cell death [81]. AKI-associated electrolyte disturbances may propagate across a disrupted BBB, especially hyponatremia, contributing to generalized cerebral edema. Finally, a key mechanism impairing cognitive function in critically ill patients is the decreased clearance of neurotoxic drugs. Unlike antibiotic-associated neurotoxicity (e.g., Cefepime) [82], which is widely recognized and routinely considered in clinical practice, the neurotoxic effects of other commonly used drugs in critically ill patients, such as opioids, gabapentinoids, and antivirals may be less readily recognized [83–85]. Their clinical manifestations may be mistaken for uremic or septic encephalopathy or even for progression of the underlying infection, as exemplified by aciclovir neurotoxicity being misinterpreted as worsening herpes encephalitis [86].

Consistent with the mechanisms observed in other organ systems, preclinical models of AKI have demonstrated that systemic inflammation compromises the BBB integrity [87]. This increase in permeability allows various molecules that are typically excluded from the central nervous system to cross the BBB. Cytokines and inflammatory mediators can activate astrocytes and glial cells, amplifying local neuroinflammation [81, 87]. Uremic toxins may activate resident microglia which, upon pathological stimulation, promote inflammation and aberrant synaptic pruning. Additionally, AKI seems to downregulate organic anion transporter 3 on the basolateral membrane of the BBB, impairing clearance of toxic metabolites and drugs from the brain [78].

#### Clinical implications

The kidney-brain interaction contributes substantially to morbidity and mortality [71, 73, 78]. Patients with AKI-associated delirium require significantly higher rates of mechanical ventilation, vasopressor support, and KRT, and exhibit increased mortality compared to non-delirious AKI patients [73]. Additionally, AKI is associated with an increased long-term risk of stroke, post-stroke mortality, and dementia. However, the interpretation of these findings is constrained by the predominantly retrospective nature of current evidence [71].

From a therapeutic perspective, frequent neurological assessment to promptly identify changes in consciousness, together with regular reassessment and optimisation of treatment strategies, represents a key component of management, including during KRT. Two post hoc analyses have suggested a potential role for KRT in mitigating the association between AKI and acute neurological dysfunction [69, 78], including delirium. However, further prospective studies are required to determine whether KRT can directly influence neurological outcomes in patients with AKI.

### Heart

The bidirectional interaction between the heart and kidneys has long been recognized as the cardiorenal syndrome (CRS) (Fig. 2) [88]. For the purpose of this review we will focus on type 3 CRS, where AKI precipitates acute cardiac dysfunction. Reported incidence of CRS type 3 varies widely, ranging from 0% to 29% [89]. In cohorts strictly excluding pre-existing cardiac comorbidities, the incidence reached 29% [90], whereas in unselected AKI cohorts lower rates were reported (17.1%) [91]. This marked heterogeneity, compounded by varying AKI definitions and the lack of specific biomarkers to distinguish AKI-induced cardiac injury from pre-existing conditions, precludes a reliable pooled incidence estimate [89].

#### Consequences of AKI

Fluid overload secondary to AKI can be particularly detrimental to the heart, especially when cardiac function is already impaired [92]. By increasing preload and ventricular filling pressures, fluid overload can impair myocardial contractility and reduce CO. Concurrently, the elevation of pulmonary pressures may lead to the development of pulmonary edema particularly where vascular leak is present such as complicating sepsis [55]. Early in this process, the RAAS and sympathetic nervous system are activated as compensatory mechanisms by both organs to maintain preload and afterload. By increasing sodium and water retention, peripheral vasoconstriction, and adrenergic tone, these mechanisms might actually worsen organ dysfunction. The ensuing venous congestion, in addition to the reduced CO, further reduces renal filtration capacity and delays renal recovery [93–95]. Interestingly, KRT may further challenge cardiac function by exacerbating preload-dependent reductions in CO following aggressive fluid removal, which may occur even in the absence of hypotension [96]. Metabolic acidosis also promotes cardiac dysfunction [97]. By reducing calcium sensitivity of contractile proteins it impairs myocardial contractility and by disrupting electrolytes transport across cardiomyocytes it fosters cardiac arrhythmias. Electrolyte disturbances associated with AKI, hyperkalemia in particular, are also relevant in this regard [98]. In addition, acidosis promotes pulmonary vasoconstriction, which may precipitate acute right ventricular dysfunction. Finally, preclinical studies have shown the contribution of uremic toxins to myocardial injury, inflammation, and remodeling [99, 100], but their role in humans remains to be investigated. Nevertheless, the precise pathogenesis of CRS type 3 has not yet been fully elucidated.

In preclinical models of AKI, elevated circulating levels of cytokines — specifically IL-1β, TNF-α and IL-6 — have been observed to exert a cardiodepressant effect [101]. By inducing cardiomyocyte apoptosis, leukocyte infiltration, oxidative stress and mitochondrial dysfunction, these mediators lead to a significant reduction in myocardial contractility [99]. Mitochondrial injury appears to be particularly relevant in this context, given the continuous ATP demand required for cardiomyocyte contraction and excitation-contraction coupling. Future research is required to evaluate whether these mechanisms could serve as novel therapeutic targets for enhancing myocardial recovery, independently of the contribution of KRT on traditional complications.

#### Clinical implications

Clinically, type 3 CRS manifests across distinct scenarios depending on the predominant pathophysiological driver [88, 99]: oliguric AKI with progressive fluid overload presenting as acute congestive heart failure and cardiogenic pulmonary edema; severe hyperkalemia or acid-base derangements triggering brady- or tachyarrhythmias; metabolic acidosis impairing myocardial contractility while blunting catecholamine responsiveness in critically ill patients. Beyond these acute short-term life-threatening events, AKI serves as an independent marker of long-term cardiovascular vulnerability. A recent systematic review and meta-analysis of 54 studies showed that AKI is associated with significantly increased risks of major adverse cardiovascular events, heart failure, myocardial infarction, stroke, and cardiovascular mortality, with excess risk evident even after stage 1 AKI [102].

Management of CRS type 3 requires an individualized approach that accounts for both the severity of AKI and the nature of the accompanying cardiac manifestation. Because acute heart failure with cardiogenic pulmonary edema appears to be the most frequent presentation [89], optimizing fluid balance, through loop diuretics or extracorporeal ultrafiltration when indicated, represents a central therapeutic objective [89, 99].

### Liver

The liver and kidneys are the two principal organ systems responsible for detoxification and maintaining metabolic homeostasis. While kidney dysfunction secondary to hepatic failure —most notably hepatorenal syndrome—is a well-recognized, albeit incompletely understood, clinical entity, the converse relationship has received considerably less attention (Fig. 2). A prospective study of critically ill patients with AKI reported that approximately 28% of them subsequently developed hepatic dysfunction, which was associated with increased mortality [103]. However, contemporary evidence on this bidirectional organ interaction remains limited.

#### Consequences of AKI

As highlighted throughout this review, fluid overload in oligo-anuric AKI is a major determinant of distant organ injury. Hepatic dysfunction resulting from AKI-associated fluid overload may be mediated by acute heart failure, placing this interaction within the framework of type 3 CRS [104]. The underlying mechanisms include hepatic venous congestion secondary to elevated central venous pressure in right-sided heart failure and hepatocellular ischemia caused by reduced CO [105]. Beyond concomitant cardiac dysfunction, a prospective study of critically ill patients without cirrhosis demonstrated that fluid overload resulting from kidney failure and aggressive fluid resuscitation, together with increasing central venous pressure, were the principal determinants of elevated liver stiffness during ICU admission. In this acute setting, liver stiffness measured by transient elastography reflects hepatic congestion rather than structural fibrosis [106].

Beyond these hemodynamic mechanisms, experimental studies have shown that AKI can directly induce sterile hepatic inflammation [104, 107]. Increased circulating concentrations of proinflammatory cytokines, particularly IL-1, IL-6, and TNF-α, activate resident Kupffer cells, initiating a secondary intrahepatic inflammatory cascade [107, 108]. This response increases hepatic vascular permeability, promotes interstitial edema, and facilitates neutrophil and lymphocyte infiltration [108]. Oxidative stress further contributes to AKI-induced liver injury. In experimental models of renal ischemia–reperfusion injury, pre-treatment with glutathione significantly attenuated hepatic injury and reduced the subsequent rise in serum transaminase concentrations, underscoring the pathogenic role of reactive oxygen species [107].

In addition to impairing renal drug clearance, AKI alters hepatic drug metabolism. The systemic accumulation of proinflammatory cytokines, reactive oxygen species, and uremic toxins during AKI suppresses the activity of hepatic cytochrome P450 enzymes [104] which are responsible for the metabolism of most xenobiotics. Consequently, reduced hepatic metabolic capacity may result in unexpected drug accumulation and toxicity, particularly for medications with a narrow therapeutic index despite preserved liver function [104, 109].

#### Clinical implications

Although the mechanisms underlying their interaction remain unclear, the coexistence of hepatic dysfunction and AKI is associated with worse outcomes compared with patients presenting with AKI alone [110]. To date, no targeted therapeutic strategies exist specifically for AKI-induced hepatic dysfunction. Management relies on fundamental principles of organ support, including volume control, hemodynamic optimization to support cardiac function, and rigorous therapeutic drug monitoring to prevent toxicity. Regarding advanced interventions, extracorporeal hemoadsorption techniques aimed at mitigating the systemic cytokine burden represent a biologically plausible rationale; however, robust clinical evidence supporting their routine use in this specific context remains insufficient [111–113].

### Immune system

Under physiological conditions, the kidney acts as an immunological organ contributing to immune homeostasis [114, 115]. Following kidney injury, an inflammatory cascade is initiated in the kidneys [116, 117]. As shown in preclinical models, this process amplifies locally and may propagate systemically, defining a possible new mechanism of distant organ damage induced by AKI (Fig. 1), including profound dysregulation of the immune system itself (Fig. 2) [118]. Clinically, AKI markedly increases patient frailty and susceptibility to de novo infections across diverse surgical and non-surgical populations [119–121]. Among AKI patients at ICU admission, de novo infections subsequently developed in 44% of those with AKI compared with 20% of patients without AKI, with the risk increasing in parallel with AKI severity [122].

#### Consequences of AKI

AKI may modulate the immune system in two different ways, including both hyperinflammation characterized by early, context-dependent activation of innate immune system, systemic cytokine release, and heightened inflammatory signaling [117] and immunoparalysis [123, 124] that mainly affects the innate immune system. Specifically, neutrophil function, including migration and phagocytosis, have been reported to be inhibited in the context of AKI [118]. This impairment could be partly mediated by resistin, an inflammatory cytokine and uremic toxin whose levels are significantly increased during AKI [125]. In patients with septic shock and AKI, neutrophil migration was reported to be more extensively suppressed compared to patients only with septic shock [125]. This immunosuppressive shift also involves antigen-presenting cell dysfunction: monocyte HLA-DR expression, a recognized marker of immunoparalysis, has been reported to be significantly lower in septic patients with AKI than in those without, independently of illness severity, mirroring a deeper state of immune depression associated with worse outcomes [126]. Conversely, when AKI occurs in a non-inflammatory setting – as mainly shown in preclinical models - the proinflammatory effect is prevalent [60, 87, 123].

#### Clinical implications

In the short term, sepsis represents a leading cause of death in critically ill patients with AKI [127]. This heightened vulnerability extends beyond the acute phase: survivors of dialysis-requiring AKI exhibit a 50%–70% higher incidence of severe sepsis compared to matched controls [120]. Crucially, this excess risk persists even after complete functional kidney recovery, indicating that AKI induces sustained immune dysregulation rather than a transient disturbance [120].

Beyond established infection prevention measures and timely, targeted antimicrobial therapy, no upstream interventions are currently available to specifically reverse AKI-associated immune dysregulation. Although hemoadsorption has demonstrated the potential to modulate elevated resistin concentrations and remove circulating inflammatory mediators in patients with septic shock, current evidence remains insufficient to support its routine clinical use in this setting [128]. Novel immunomodulatory approaches targeting maladaptive inflammatory pathways are under investigation, including recombinant CD39 (TIN816) as a potential therapeutic strategy in sepsis-associated AKI (CLEAR-AKI, NCT05996835).

Based on the aforementioned organ-crosstalks between the kidneys and other organs, the resulting organ dysfunction(s) and complications might be considered as endpoints in AKI trials. The following section considers how these consequences can inform the selection of primary and surrogate endpoints in AKI trials, and why traditional long-term kidney-centric metrics such as MAKE may not capture the full burden of the syndrome in non-enriched populations.

## AKI trial endpoints: surrogate markers of AKI-associated complications

In considering optimal AKI trial endpoints, the purpose of the trial impacts endpoint options. AKI prevention and attenuation trials endpoints should ideally account for the incidence, severity, and duration of AKI allowing for gradation across the spectrum of AKI. Long-term outcomes (e.g. Major Adverse Kidney Events —MAKE—death, initiation of dialysis, and persistent kidney dysfunction) are not ideally suited as their incidence in non-enriched populations in prevention trials will be extremely low (< 5–10%). Thus, using this outcome would necessitate trials of several thousand patients to demonstrate efficacy [129]. Treatment trials should utilize patient-centered long-term outcomes (e.g. MAKE, hospital and dialysis free days) although depending on the cohort, clinical setting, and intervention, one component of MAKE may dominate the overall outcomes.

Importantly, when considering AKI as an outcome for a trial, short-term and transient changes in creatinine - and urine output-based endpoints are practical and will undoubtedly increase event rates, facilitating smaller and faster trials. However, it remains unclear how small changes in serum creatinine reliably predict long-term outcomes. While some epidemiology studies demonstrate links between Stage 1 AKI and adverse outcomes, the data is inconsistent [130–132]. However trials performed in critically ill patients, are different from those looking at community-acquired or ward-based AKI, and ICU investigations are better suited to look at more severe and persistent AKI and MAKE.

Furthermore, MAKE, especially mortality, is influenced by factors unrelated to AKI (e.g. post-operative sepsis or stroke), and mortality is a competing risk for long-term outcomes such as CKD and dialysis-dependence/ESKD [133]. Strategies for investigating CKD progression as an outcome after AKI include: ensuring adequate proportions of patients in each stage of AKI severity, matching patients based on age, baseline kidney function, and comorbidities, and frequent laboratory follow-up to analyze trajectory of kidney function [134]. Importantly, establishing baseline (pre-enrolment) kidney function can be problematic, depending on the availability of prior serum creatinine data, and this can impact AKI incidence, AKI severity, and the incidence of recovery. In patients without known CKD, when a reliable premorbid creatinine (or cystatin C) value is unavailable, the 2012 KDIGO guideline recommends estimating baseline creatinine either by back-calculation using the Modification of Diet in Renal Disease equation (assuming a baseline eGFR of 75 mL/min/1.73 m²) or using the lowest in-hospital creatinine value as a surrogate, provided unrecognized CKD has been excluded [15]. However, back calculation can both over and under estimate the incidence of AKI depending on the cohort. It is more likely to underestimate the incidence in younger healthy cohorts and over estimates in sicker and older cohorts [135, 136]. To overcome these issues, albuminuria may be used as a surrogate marker of CKD progression. The prospective, longitudinal, observational ASSESS-AKI study found that higher urine albumin-creatinine ratio 3 months after hospitalization with AKI was associated with increased risk of CKD progression (HR 1.53, 95% CI 1.45–1.62) [137].

Albuminuria could serve as a surrogate to predict patients at risk of CKD progression after AKI as well as a therapeutic target, such as with renin-angiotensin-aldosterone inhibitors, sodium-glucose cotransporter 2 inhibitors (SGLT2i) or glucagon-like peptide-1 receptor agonist (GLP-1) [137–142]. Other structural and functional biomarkers, including plasma soluble tumor necrosis factor receptor 1 and 2 (sTNFR1, sTNFR2), urine neutrophil gelatinase-associated lipocalin (uNGAL), urinary tissue inhibitor of metalloproteinase 2 and insulin-like growth factor–binding protein 7 (TIMP-2*IGFBP7), have been shown to predict CKD progression or recovery from AKI and may have a role as a surrogate outcome in AKI trials, however this requires future investigation and validation [143–149]. This is separate from the use of damage and stress biomarkers as an enrichment strategy to increase AKI incidence and severity [150–154].

In trials of patients with AKI who require dialysis, standardized definitions of recovery and dialysis dependence are lacking; as such, several definitions have characterized renal recovery or dialysis dependence including continued dialysis at hospital discharge, 28 days, and/or 90 days [155–157]. Dialysis dependence at 90 days is consistent with the definition of ESKD, and while this is certainly a patient-centered outcome, assessment before 90-days is equally relevant. Among these trials, definitions of recovery from dialysis dependence also vary, such as being alive without dialysis for 14 consecutive days as used in the LIBERATE-D trial, or lack of need for dialysis with a minimal creatinine clearance of 20 ml/min used in a post hoc analysis of the Acute Renal Failure Trial Network (ATN) study [155, 156]. Days alive and dialysis-free is a novel alternative endpoint that accounts for a patient-centered outcome as well as mortality associated with in-hospital AKI [155, 158–162]. However, this is inherently a composite outcome that treats death and dialysis dependence as equally important. Hierarchical composite endpoints and calculations of win ratios have been explored to account for the relative importance of different outcomes and provide more nuanced interpretations of results of AKI trials where death and dialysis-dependence are competing outcomes [133, 163]. Even among patients who no longer require dialysis after AKI, the risk of recurrent AKI and development of ESKD remains high, and future studies should investigate how to optimize care after incident AKI [164].

Systemic complications from AKI are often included in secondary, post hoc, or safety outcomes of AKI trials. Examples of systemic complications include prolonged mechanical ventilation, cardiac arrhythmias, exposure to hypotension / hemodynamic instability, delirium, infections, and bleeding. Table 3 describes outcomes from AKI trials related to other organ systems. Since AKI is associated with these complications and these influence patient outcome, these complications can also be used as a composite endpoint in treatment trials.

Finally, given the high morbidity associated with hospital-acquired AKI, it is important for AKI trials to increase and expand the measurement of patient- and caregiver- centered outcomes for those who survive their critical illness. Length of stay and readmission rates, while often regarded as hospital quality metrics, also represent time patients spend in the hospital rather than at home and can be a useful outcome in AKI prevention and post-discharge AKI care trials [150, 165–167]. However, this endpoint should only be used in a context-specific manner (e.g. in the context of sepsis-associated AKI, but not for trials investigating cardiac surgery associated AKI). This endpoint should also differentiate between time at a non-hospital facility (e.g. skilled nursing facility or long-term acute care facility) and time at home.

Financial burden and impact on employment are equally life-changing patient-centered outcomes, but are seldom explored in AKI studies [158]. Health-related quality of life and physical function have frequently been shown to decrease after hospitalization with AKI [168–170]. Trials surrounding dialysis initiation for AKI, such as STARRT-AKI and AKIKI2 trials, have thus included measures of quality of life and activities of daily living as secondary outcomes [159–161]. To optimize post-ICU physical function, some studies have explored the feasibility and effects of early mobilization while on continuous kidney replacement therapy (CKRT) [171–173]. Future AKI trials should incorporate outcomes that pertain to patients’ and caregivers’ perceptions and experiences during and after hospitalization with AKI.

**Table 3:** Novel endpoints for AKI trials

Outcome	Interaction with AKI	Examples from prior trials
Mechanical ventilation/Ventilator-Free Days	Fluid overload can lead to or exacerbate pulmonary edema, hypoxemia, and decreased lung compliance	Delayed initiation or conservative dialysis strategies do not appear to increase days on mechanical ventilation, with the caveat that protocols allowed for dialysis per clinician judgement [155, 161, 162, 174]
	Hypophosphatemia from prolonged CKRT can contribute to neuromuscular weakness	Hypophosphatemia during CKRT was associated with lower chance of successful extubation [175, 176] and fewer ventilator-free days [177]
Cardiac arrhythmias	Electrolyte abnormalities related to AKI can precipitate cardiac arrhythmias	Delayed initiation or conservative dialysis strategies do not appear to increase incidence of cardiac arrhythmias [155, 161]
Hemodynamics	Volume balance and dialysis can impact the need for vasoactive medications	Intermittent KRT modalities were associated with hypotension and/or cardiac arrhythmias during KRT [178]
		Accelerated or delayed dialysis initiation strategies were not associated with vasopressor-free days [159, 161]
Delirium/Coma	Accumulation of uremic toxins or neurotoxic medications/metabolites can contribute to delirium/coma	A more-delayed dialysis initiation strategy was associated with a lower chance of awakening among comatose patients with AKI [78]
Infections	Placement of dialysis catheters could increase risk for bloodstream infections	An accelerated dialysis initiation strategy was not associated with catheter-related bloodstream infections [159]
	Clearance by dialysis can affect antimicrobial drug levels	Patients on CKRT experience high rates of multi-drug resistant infections [179]
Postoperative bleeding	Accumulation of uremic toxins and fluid overload could contribute to bleeding after surgery	Postoperative AKI has been associated with increased risk of bleeding complications [180, 181]

AKI= Acute kidney ijury; CKRT= Continuous kidney replacement therapy; KRT = Kidney replacement therapy

## Conclusions

Although the molecular mechanisms are not fully elucidated, the literature consistently reports the association between the failing kidneys and other organ dysfunctions and that AKI-associated complications are associated with an increased morbidity and mortality. Unfortunately, no therapies directly targeting AKI exist; therefore, physicians can only apply preventive and supportive measures. As these AKI-associated complications directly influence the outcome of the patients, they may be considered as endpoints in AKI trials.

## Data Availability

No datasets were generated or analysed during the current study.

## Abbreviations

AKI: Acute Kidney Injury
ALI: Acute Lung Injury
ARDS: Acute Respiratory Distress Syndrome
BBB: Blood Brain Barrier
BUN: Blood Urea Nitrogen
CKD: Chronic Kidney Disease
CO: Cardiac Output
CKRT: Continuous Kidney Replacement Therapy
CRS: Cardiorenal Syndrome
DAMPs: Damage Associated Molecular Patterns
ESKD: End Stage Kidney Disease
GFR: Glomerular Filtration Rate
GLP-1: Glucagon-like Peptide 1 receptor agonist
HMGB-1: High Mobility Group Box 1
ICU: Intensive Care Unit
IGFBP7: Insulin-like Growth Factor–Binding Protein 7
IL: Interleukin
KDIGO: Kidney Disease: Improving Global Outcomes
KRT: Kidney Replacement Therapy
LOS: Length of Stay
MAKE: Major Adverse Kidney Events
MRI: Magnetic Resonance Imaging
PRES: Posterior Reversible Encephalopathy Syndrome
RAAS: Renin-Angiotensin-Aldosterone System
SGLT2i: Sodium-Glucose Cotransporter 2 inhibitors
sTNFR1: Soluble Tumor Necrosis Factor Receptor 1
sTNFR2: Soluble Tumor Necrosis Factor Receptor 2
TIMP2: Tissue Inhibitor of Metalloproteinase 2
TLR: Toll-like receptor
TNF-α: Tumor Necrosis Factor alpha
uNGAL: Urine Neutrophil Gelatinase-Associated Lipocalin

## References

[1] Mehta RL, Burdmann EA, Cerdá J, Feehally J, Finkelstein F, García-García G, et al. Recognition and management of acute kidney injury in the International Society of Nephrology 0by25 Global Snapshot: a multinational cross-sectional study. Lancet. 2016;387:2017–25. 10.1016/S0140-6736(16)30240-9. 27086173 10.1016/S0140-6736(16)30240-9

[2] Hoste EAJ, Kellum JA, Selby NM, Zarbock A, Palevsky PM, Bagshaw SM, et al. Global epidemiology and outcomes of acute kidney injury. Nat Rev Nephrol. 2018;14:607–25. 10.1038/s41581-018-0052-0. 30135570 10.1038/s41581-018-0052-0

[3] Fujii T, Uchino S, Doi K, Sato T, Kawamura T. Diagnosis, management, and prognosis of patients with acute kidney injury in Japanese intensive care units: The JAKID study. J Crit Care. 2018;47:185–91. 10.1016/j.jcrc.2018.07.007. 30015288 10.1016/j.jcrc.2018.07.007

[4] Al-Jaghbeer M, Dealmeida D, Bilderback A, Ambrosino R, Kellum JA. Clinical Decision Support for In-Hospital AKI. J Am Soc Nephrol. 2018;29:654–60. 10.1681/ASN.2017070765. 29097621 10.1681/ASN.2017070765 PMC5791078

[5] Hoste EAJ, Bagshaw SM, Bellomo R, Cely CM, Colman R, Cruz DN, et al. Epidemiology of acute kidney injury in critically ill patients: the multinational AKI-EPI study. Intensive Care Med. 2015;41:1411–23. 10.1007/s00134-015-3934-7. 26162677 10.1007/s00134-015-3934-7

[6] Sawhney S, Marks A, Fluck N, Levin A, McLernon D, Prescott G, et al. Post-discharge kidney function is associated with subsequent ten-year renal progression risk among survivors of acute kidney injury. Kidney Int. 2017;92:440–52. 10.1016/j.kint.2017.02.019. 28416224 10.1016/j.kint.2017.02.019 PMC5524434

[7] See EJ, Jayasinghe K, Glassford N, Bailey M, Johnson DW, Polkinghorne KR, et al. Long-term risk of adverse outcomes after acute kidney injury: a systematic review and meta-analysis of cohort studies using consensus definitions of exposure. Kidney Int. 2019;95:160–72. 10.1016/j.kint.2018.08.036. 30473140 10.1016/j.kint.2018.08.036

[8] Parr SK, Matheny ME, Abdel-Kader K, Greevy RA, Bian A, Fly J, et al. Acute kidney injury is a risk factor for subsequent proteinuria. Kidney Int. 2018;93:460–9. 10.1016/j.kint.2017.07.007. 28927644 10.1016/j.kint.2017.07.007 PMC5796522

[9] Jones J, Holmen J, De Graauw J, Jovanovich A, Thornton S, Chonchol M. Association of complete recovery from acute kidney injury with incident CKD stage 3 and all-cause mortality. Am J Kidney Dis Off J Natl Kidney Found. 2012;60:402–8. 10.1053/j.ajkd.2012.03.014. 10.1053/j.ajkd.2012.03.014 PMC3422603 22541737

[10] Heung M, Steffick DE, Zivin K, Gillespie BW, Banerjee T, Hsu C, et al. Acute Kidney Injury Recovery Pattern and Subsequent Risk of CKD: An Analysis of Veterans Health Administration Data. Am J Kidney Dis. 2016;67:742–52. 10.1053/j.ajkd.2015.10.019. 26690912 10.1053/j.ajkd.2015.10.019 PMC6837804

[11] De Mendonça A, Vincent J-L, Suter PM, Moreno R, Dearden NM, Antonelli M, et al. Acute renal failure in the ICU: risk factors and outcome evaluated by the SOFA score. Intensive Care Med. 2000;26:915–21. 10.1007/s001340051281. 10990106 10.1007/s001340051281

[12] Russell JA, Singer J, Bernard GR, Wheeler A, Fulkerson W, Hudson L, et al. Changing pattern of organ dysfunction in early human sepsis is related to mortality. Crit Care Med. 2000;28:3405–11. 10.1097/00003246-200010000-00005. 11057793 10.1097/00003246-200010000-00005

[13] Clermont G, Acker CG, Angus DC, Sirio CA, Pinsky MR, Johnson JP. Renal failure in the ICU: Comparison of the impact of acute renal failure and end-stage renal disease on ICU outcomes. Kidney Int. 2002;62:986–96. 10.1046/j.1523-1755.2002.00509.x. 12164882 10.1046/j.1523-1755.2002.00509.x

[14] Zarbock A, Weiss R, Albert F, Rutledge K, Kellum JA, Bellomo R, et al. Epidemiology of surgery associated acute kidney injury (EPIS-AKI): a prospective international observational multi-center clinical study. Intensive Care Med. 2023;49:1441–55. 10.1007/s00134-023-07169-7. 37505258 10.1007/s00134-023-07169-7 PMC10709241

[15] Kidney Disease. Improving Global Outcomes (KDIGO) Acute Kidney Injury Work Group. KDIGO Clinical Practice Guideline for Acute Kidney Injury. Volume 2. Kidney inter.; 2012:1–138.

[16] Lau WL, Vaziri ND. Urea, a true uremic toxin: the empire strikes back. Clin Sci. 2017;131:3–12. 10.1042/CS20160203. 10.1042/CS20160203 27872172

[17] Neirynck N, Vanholder R, Schepers E, Eloot S, Pletinck A, Glorieux G. An update on uremic toxins. Int Urol Nephrol. 2013;45:139–50. 10.1007/s11255-012-0258-1. 22893494 10.1007/s11255-012-0258-1

[18] Jaber S, Paugam C, Futier E, Lefrant J-Y, Lasocki S, Lescot T, et al. Sodium bicarbonate therapy for patients with severe metabolic acidaemia in the intensive care unit (BICAR-ICU): a multicentre, open-label, randomised controlled, phase 3 trial. Lancet. 2018;392:31–40. 10.1016/S0140-6736(18)31080-8. 29910040 10.1016/S0140-6736(18)31080-8

[19] Malbrain MLNG, Martin G, Ostermann M. Everything you need to know about deresuscitation. Intensive Care Med. 2022;48:1781–6. 10.1007/s00134-022-06761-7. 35932335 10.1007/s00134-022-06761-7 PMC9362613

[20] Lowe KG, THE LATE PROGNOSIS IN ACUTE TUBULAR NECROSIS. Lancet. 1952;259:1086–8. 10.1016/S0140-6736(52)90744-7. 10.1016/s0140-6736(52)90744-7 14928581

[21] Chawla LS, Kimmel PL. Acute kidney injury and chronic kidney disease: an integrated clinical syndrome. Kidney Int. 2012;82:516–24. 10.1038/ki.2012.208. 22673882 10.1038/ki.2012.208

[22] Ishani A, Nelson D, Clothier B, Schult T, Nugent S, Greer N, et al. The Magnitude of Acute Serum Creatinine Increase After Cardiac Surgery and the Risk of Chronic Kidney Disease, Progression of Kidney Disease, and Death. Arch Intern Med. 2011;171:226. 10.1001/archinternmed.2010.514. 21325112 10.1001/archinternmed.2010.514

[23] Thakar CV, Christianson A, Himmelfarb J, Leonard AC. Acute Kidney Injury Episodes and Chronic Kidney Disease Risk in Diabetes Mellitus. Clin J Am Soc Nephrol. 2011;6:2567–72. 10.2215/CJN.01120211. 21903988 10.2215/CJN.01120211 PMC3359576

[24] Coca SG, Singanamala S, Parikh CR. Chronic kidney disease after acute kidney injury: a systematic review and meta-analysis. Kidney Int. 2012;81:442–8. 10.1038/ki.2011.379. 22113526 10.1038/ki.2011.379 PMC3788581

[25] Ishani A, Xue JL, Himmelfarb J, Eggers PW, Kimmel PL, Molitoris BA, et al. Acute Kidney Injury Increases Risk of ESRD among Elderly. J Am Soc Nephrol. 2009;20:223–8. 10.1681/ASN.2007080837. 19020007 10.1681/ASN.2007080837 PMC2615732

[26] Wald R. Chronic Dialysis and Death Among Survivors of Acute Kidney Injury Requiring Dialysis. JAMA. 2009;302:1179. 10.1001/jama.2009.1322. 19755696 10.1001/jama.2009.1322

[27] Basile DP, Donohoe D, Roethe K, Osborn JL. Renal ischemic injury results in permanent damage to peritubular capillaries and influences long-term function. Am J Physiol Ren Physiol. 2001;281:F887–899. 10.1152/ajprenal.2001.281.5.F887. 10.1152/ajprenal.2001.281.5.F887 11592947

[28] Venkatachalam MA, Griffin KA, Lan R, Geng H, Saikumar P, Bidani AK. Acute kidney injury: a springboard for progression in chronic kidney disease. Am J Physiol Ren Physiol. 2010;298:F1078–1094. 10.1152/ajprenal.00017.2010. 10.1152/ajprenal.00017.2010 PMC2867413 20200097

[29] Chawla LS, Eggers PW, Star RA, Kimmel PL. Acute kidney injury and chronic kidney disease as interconnected syndromes. N Engl J Med. 2014;371:58–66. 10.1056/NEJMra1214243. 24988558 10.1056/NEJMra1214243 PMC9720902

[30] Bellomo R, Kellum JA, Ronco C. Defining acute renal failure: physiological principles. Intensive Care Med. 2004;30:33–7. 10.1007/s00134-003-2078-3. 14618231 10.1007/s00134-003-2078-3

[31] Pickkers P, Darmon M, Hoste E, Joannidis M, Legrand M, Ostermann M, et al. Acute kidney injury in the critically ill: an updated review on pathophysiology and management. Intensive Care Med. 2021;47:835–50. 10.1007/s00134-021-06454-7. 34213593 10.1007/s00134-021-06454-7 PMC8249842

[32] Andrade-Oliveira V, Foresto-Neto O, Watanabe IKM, Zatz R, Câmara NOS. Inflammation in Renal Diseases: New and Old Players. Front Pharmacol. 2019;10:1192. 10.3389/fphar.2019.01192. 31649546 10.3389/fphar.2019.01192 PMC6792167

[33] Bechtel W, McGoohan S, Zeisberg EM, Müller GA, Kalbacher H, Salant DJ, et al. Methylation determines fibroblast activation and fibrogenesis in the kidney. Nat Med. 2010;16:544–50. 10.1038/nm.2135. 20418885 10.1038/nm.2135 PMC3106179

[34] Kawakami T, Ren S, Duffield JS. Wnt signalling in kidney diseases: dual roles in renal injury and repair. J Pathol. 2013;229:221–31. 10.1002/path.4121. 23097132 10.1002/path.4121

[35] Yang L, Besschetnova TY, Brooks CR, Shah JV, Bonventre JV. Epithelial cell cycle arrest in G2/M mediates kidney fibrosis after injury. Nat Med. 2010;16:535–43. 10.1038/nm.2144. 1p following 143. 20436483 10.1038/nm.2144 PMC3928013

[36] Schrimpf C, Duffield JS. Mechanisms of fibrosis: the role of the pericyte. Curr Opin Nephrol Hypertens. 2011;20:297–305. 10.1097/MNH.0b013e328344c3d4. 21422927 10.1097/MNH.0b013e328344c3d4

[37] Eddy AA. The origin of scar-forming kidney myofibroblasts. Nat Med. 2013;19:964–6. 10.1038/nm.3299. 23921738 10.1038/nm.3299

[38] Bonventre JV, Basile D, Liu KD, McKay D, Molitoris BA, Nath KA, et al. AKI: a path forward. Clin J Am Soc Nephrol CJASN. 2013;8:1606–8. 10.2215/CJN.06040613. 23868899 10.2215/CJN.06040613 PMC3805072

[39] Prud’homme M, Coutrot M, Michel T, Boutin L, Genest M, Poirier F, et al. Acute Kidney Injury Induces Remote Cardiac Damage and Dysfunction Through the Galectin-3 Pathway. JACC Basic Transl Sci. 2019;4:717–32. 10.1016/j.jacbts.2019.06.005. 31709320 10.1016/j.jacbts.2019.06.005 PMC6834958

[40] Jabaudon M, Blondonnet R, Ware LB. Biomarkers in acute respiratory distress syndrome. Curr Opin Crit Care. 2021;27:46–54. 10.1097/MCC.0000000000000786. 33278123 10.1097/MCC.0000000000000786 PMC7774234

[41] Rühlmann M, Xu L, Bauer M, Lehmann T, Panagiotou G, Neugebauer S, et al. Correlation of brain injury biomarkers with brain dysfunction, brain injury, and outcomes in critically ill patients: a post hoc exploratory analysis. Infection. 2026;54:1501–17. 10.1007/s15010-026-02790-2. 41973367 10.1007/s15010-026-02790-2 PMC13323603

[42] Duda I, Krzych Ł, Jędrzejowska-Szypułka H, Lewin-Kowalik J. Serum levels of the S100B protein and neuron-specific enolase are associated with mortality in critically ill patients. Acta Biochim Pol. 2017;64:647–52. 10.18388/abp.2017_1619. 29222857 10.18388/abp.2017_1619

[43] Rangaswami J, Bhalla V, Blair JEA, Chang TI, Costa S, Lentine KL, et al. Cardiorenal Syndrome: Classification, Pathophysiology, Diagnosis, and Treatment Strategies: A Scientific Statement From the American Heart Association. Circulation [Internet]. 2019. 10.1161/CIR.0000000000000664. [cited 2026 Jul 25];139. 30852913 10.1161/CIR.0000000000000664

[44] Geavlete O, Ambrosy AP, Mebazaa A, Collins SP, Biegus J, Adamo M, et al. Liver Function Abnormalities in Acute Heart Failure: A State-of-the-art Review from an International Expert Group. Card Fail Rev. 2026;e17. 10.15420/cfr.2025.51. 10.15420/cfr.2025.51 PMC13392301 42495596

[45] Fu S, Wu D, Jiang W, Li J, Long J, Jia C, et al. Molecular Biomarkers in Drug-Induced Liver Injury: Challenges and Future Perspectives. Front Pharmacol. 2020;10:1667. 10.3389/fphar.2019.01667. 32082163 10.3389/fphar.2019.01667 PMC7002317

[46] Edwin E, Jayaprakash N. Inflammation-driven multiorgan Dysfunction: Biomarker-Guided diagnosis, mechanistic insights and emerging precision therapeutic strategies. Adv Biomark Sci Technol. 2027;9:1–25. 10.1016/j.abst.2026.06.002.

[47] Ostermann M, Legrand M, Meersch M, Srisawat N, Zarbock A, Kellum JA. Biomarkers in acute kidney injury. Ann Intensive Care. 2024;14:145. 10.1186/s13613-024-01360-9. 39279017 10.1186/s13613-024-01360-9 PMC11402890

[48] Alge J, Dolan K, Angelo J, Thadani S, Virk M, Akcan Arikan A. Two to Tango: Kidney-Lung Interaction in Acute Kidney Injury and Acute Respiratory Distress Syndrome. Front Pediatr. 2021;9:744110. 10.3389/fped.2021.744110. 34733809 10.3389/fped.2021.744110 PMC8559585

[49] Rocşoreanu A, Cernea D, Moţa E. The Complexity of Pulmonary Complications in Acute Kidney Injury. Curr Health Sci J. 2017;43:69–72. 10.12865/CHSJ.43.01.10. 30595857 10.12865/CHSJ.43.01.10 PMC6286720

[50] Teixeira JP, Ambruso S, Griffin BR, Faubel S. Pulmonary Consequences of Acute Kidney Injury. Semin Nephrol. 2019;39:3–16. 10.1016/j.semnephrol.2018.10.001. 30606405 10.1016/j.semnephrol.2018.10.001

[51] Kumar A, Epler K, DeWolf S, Barnes L, Hepokoski M. Bidirectional pressure: a mini review of ventilator-lung-kidney interactions. Front Physiol. 2024;15:1428177. 10.3389/fphys.2024.1428177. 38966229 10.3389/fphys.2024.1428177 PMC11222611

[52] Ronco C, Bellomo R, Kellum JA. Acute kidney injury. Lancet. London, England; 2019;394:1949–64. 10.1016/S0140-6736(19)32563-2 10.1016/S0140-6736(19)32563-2 31777389

[53] Prowle JR, Echeverri JE, Ligabo EV, Ronco C, Bellomo R. Fluid balance and acute kidney injury. Nat Rev Nephrol Nat Publishing Group. 2010;6:107–15. 10.1038/nrneph.2009.213. 10.1038/nrneph.2009.213 20027192

[54] Faubel S, Pulmonary Complications After Acute Kidney Injury. Adv Chronic Kidney Dis. 2008;15:284–96. 10.1053/j.ackd.2008.04.008. 18565479 10.1053/j.ackd.2008.04.008

[55] Gheorghiade M, Filippatos G, De Luca L, Burnett J. Congestion in Acute Heart Failure Syndromes: An Essential Target of Evaluation and Treatment. Am J Med. 2006;119:S3–10. 10.1016/j.amjmed.2006.09.011. 17113398 10.1016/j.amjmed.2006.09.011

[56] Hayashi Y, Shimazui T, Tomita K, Shimada T, Miura RE, Nakada T. Associations between fluid overload and outcomes in critically ill patients with acute kidney injury: a retrospective observational study. Sci Rep. 2023;13:17410. 10.1038/s41598-023-44778-0. 37833430 10.1038/s41598-023-44778-0 PMC10575912

[57] Vemuri SV, Rolfsen ML, Sykes AV, Takiar PG, Leonard AJ, Malhotra A, et al. Association Between Acute Kidney Injury During Invasive Mechanical Ventilation and ICU Outcomes and Respiratory System Mechanics. Crit Care Explor. 2022;4:e0720. 10.1097/CCE.0000000000000720. 35782295 10.1097/CCE.0000000000000720 PMC9246080

[58] Kusirisin P, Bagshaw SM. Kidney-ventilator interaction and kidney-protective ventilation. Curr Opin Crit Care [Internet]. 2025. 10.1097/MCC.0000000000001342. [cited 2026 Jul 24]. 41481828 10.1097/MCC.0000000000001342

[59] Hoke TS, Douglas IS, Klein CL, He Z, Fang W, Thurman JM, et al. Acute Renal Failure after Bilateral Nephrectomy Is Associated with Cytokine-Mediated Pulmonary Injury. J Am Soc Nephrol. 2007;18:155–64. 10.1681/ASN.2006050494. 17167117 10.1681/ASN.2006050494

[60] Klein CL, Hoke TS, Fang W-F, Altmann CJ, Douglas IS, Faubel S. Interleukin-6 mediates lung injury following ischemic acute kidney injury or bilateral nephrectomy. Kidney Int. 2008;74:901–9. 10.1038/ki.2008.314. 18596724 10.1038/ki.2008.314

[61] Hepokoski M, Wang J, Li K, Li Y, Gupta P, Mai T, et al. Altered lung metabolism and mitochondrial DAMPs in lung injury due to acute kidney injury. Am J Physiol-Lung Cell Mol Physiol. 2021;320:L821–31. 10.1152/ajplung.00578.2020. 33565357 10.1152/ajplung.00578.2020 PMC8174821

[62] Kim J, Baalachandran R, Li Y, Zhang C-O, Ke Y, Karki P, et al. Circulating extracellular histones exacerbate acute lung injury by augmenting pulmonary endothelial dysfunction via TLR4-dependent mechanism. Am J Physiol-Lung Cell Mol Physiol. 2022;323:L223–39. 10.1152/ajplung.00072.2022. 35852995 10.1152/ajplung.00072.2022 PMC9512107

[63] Komaru Y, Ning L, Lama C, Suresh A, Kefaloyianni E, Miller MJ, et al. Acute kidney injury triggers hypoxemia by lung intravascular neutrophil retention that reduces capillary blood flow. J Clin Invest. 2025;135:e186705. 10.1172/JCI186705. 40048367 10.1172/JCI186705 PMC12077900

[64] Rabb H, Wang Z, Nemoto T, Hotchkiss J, Yokota N, Soleimani M. Acute renal failure leads to dysregulation of lung salt and water channels. Kidney Int. 2003;63:600–6. 10.1046/j.1523-1755.2003.00753.x. 12631124 10.1046/j.1523-1755.2003.00753.x

[65] Murugan R, Kerti SJ, Chang C-CH, Gallagher M, Clermont G, Palevsky PM, et al. Association of Net Ultrafiltration Rate With Mortality Among Critically Ill Adults With Acute Kidney Injury Receiving Continuous Venovenous Hemodiafiltration: A Secondary Analysis of the Randomized Evaluation of Normal vs Augmented Level (RENAL) of Renal Replacement Therapy Trial. JAMA Netw Open. 2019;2:e195418. 10.1001/jamanetworkopen.2019.5418. 31173127 10.1001/jamanetworkopen.2019.5418 PMC6563576

[66] Tomasi A, Song X, Gajic O, Kashani K. Kidney and lung crosstalk during critical illness: large-scale cohort study. J Nephrol. 2023;36:1037–46. 10.1007/s40620-022-01558-9. 36692665 10.1007/s40620-022-01558-9

[67] Vaara ST, Ostermann M, Bitker L, Schneider A, Poli E, Hoste E, et al. Restrictive fluid management versus usual care in acute kidney injury (REVERSE-AKI): a pilot randomized controlled feasibility trial. Intensive Care Med. 2021;47:665–73. 10.1007/s00134-021-06401-6. 33961058 10.1007/s00134-021-06401-6 PMC8195764

[68] Porschen C, Strauß C, Bagshaw SM, Kellum JA, Meuth SG, Brauckmann P, et al. Association between acute kidney injury and delirium in critically ill patients: a retrospective cohort study using two independent databases. Crit Care. 2026;30:158. 10.1186/s13054-026-05922-0. 41787440 10.1186/s13054-026-05922-0 PMC13063765

[69] Siew ED, Fissell WH, Tripp CM, Blume JD, Wilson MD, Clark AJ, et al. Acute Kidney Injury as a Risk Factor for Delirium and Coma during Critical Illness. Am J Respir Crit Care Med. 2017;195:1597–607. 10.1164/rccm.201603-0476OC. 27854517 10.1164/rccm.201603-0476OC PMC5476907

[70] Rosner MH, Husain-Syed F, Reis T, Ronco C, Vanholder R. Uremic encephalopathy. Kidney Int. 2022;101:227–41. 10.1016/j.kint.2021.09.025. 34736971 10.1016/j.kint.2021.09.025

[71] Kelly DM, Kelleher EM, Rothwell PM. Acute Kidney Injury and Risk of Adverse Neurocognitive Outcomes: A Systematic Review and Meta-Analysis. Neurology. 2026;106:e218031. 10.1212/WNL.0000000000218031. 42096677 10.1212/WNL.0000000000218031 PMC13165293

[72] Neurologic complications of acute and chronic renal disease. Handb Clin Neurol [Internet]. Elsevier; 2014 [cited 2026 Jul 24]. pp. 383–93. 10.1016/B978-0-7020-4086-3.00024-2 10.1016/B978-0-7020-4086-3.00024-2 24365307

[73] Kusirisin P, Corsaro A, Kung JY, Rewa OG, Wilcox ME, Bagshaw SM. Association Between Acute Kidney Injury, Delirium, and Outcomes in Patients With Critical Illness: A Systematic Review and Meta-Analysis. Crit Care Med. 2026;54:571–83. 10.1097/CCM.0000000000006997. 41405369 10.1097/CCM.0000000000006997

[74] Tanaka S, Okusa MD. AKI and the Neuroimmune Axis. Semin Nephrol. 2019;39:85–95. 10.1016/j.semnephrol.2018.10.008. 30606410 10.1016/j.semnephrol.2018.10.008 PMC7316566

[75] Haines RW, Prowle JR, Day A, Bear DE, Heyland DK, Puthucheary Z. Association between urea trajectory and protein dose in critically ill adults: a secondary exploratory analysis of the effort protein trial (RE-EFFORT). Crit Care. London, England; 2024;28:24. 10.1186/s13054-024-04799-1 10.1186/s13054-024-04799-1 PMC10792897 38229072

[76] Chawla LS. Permissive azotemia during acute kidney injury enables more rapid renal recovery and less renal fibrosis: a hypothesis and clinical development plan. Crit Care. 2022;26:116. 10.1186/s13054-022-03988-0. 35484549 10.1186/s13054-022-03988-0 PMC9047291

[77] Adesso S, Magnus T, Cuzzocrea S, Campolo M, Rissiek B, Paciello O, et al. Indoxyl Sulfate Affects Glial Function Increasing Oxidative Stress and Neuroinflammation in Chronic Kidney Disease: Interaction between Astrocytes and Microglia. Front Pharmacol. 2017;8:370. 10.3389/fphar.2017.00370. 28659803 10.3389/fphar.2017.00370 PMC5466960

[78] Rambaud T, Hajage D, Dreyfuss D, Lebbah S, Martin-Lefevre L, Louis G, et al. Renal replacement therapy initiation strategies in comatose patients with severe acute kidney injury: a secondary analysis of a multicenter randomized controlled trial. Intensive Care Med. 2024;50:385–94. 10.1007/s00134-024-07339-1. 38407824 10.1007/s00134-024-07339-1

[79] Trinh-Trang-Tan M-M, Cartron J-P, Bankir L. Molecular basis for the dialysis disequilibrium syndrome: altered aquaporin and urea transporter expression in the brain. Nephrol Dial Transpl. 2005;20:1984–8. 10.1093/ndt/gfh877. 10.1093/ndt/gfh877 15985519

[80] Mourid MR, Oweidat M, Abady E, Shahid MA, Ata IM, Shaban NS, et al. Posterior reversible encephalopathy syndrome (PRES): A narrative review of pathophysiology, clinical insights, and advances in management. Int J Emerg Med. 2026;19:48. 10.1186/s12245-026-01138-9. 41714952 10.1186/s12245-026-01138-9 PMC12922249

[81] Nongnuch A, Panorchan K, Davenport A. Brain–kidney crosstalk. Crit Care. 2014;18:225. 10.1186/cc13907. 25043644 10.1186/cc13907 PMC4075125

[82] Radkowski P, Oszytko J, Sobolewski K, Trachte F, Onichimowski D, Majewska M. The Effect of Antibiotics on the Nervous System: Importance for Anesthesiology and Intensive Care. Antibiotics. 2025;14:622. 10.3390/antibiotics14060622. 40558212 10.3390/antibiotics14060622 PMC12189792

[83] Hellden A, Odar-Cederlof I, Diener P, Barkholt L, Medin C, Svensson J-O, et al. High serum concentrations of the acyclovir main metabolite 9-carboxymethoxymethylguanine in renal failure patients with acyclovir-related neuropsychiatric side effects: an observational study. Nephrol Dial Transpl. 2003;18:1135–41. 10.1093/ndt/gfg119. 10.1093/ndt/gfg119 12748346

[84] Lee KA, Ganta N, Horton JR, Chai E. Evidence for Neurotoxicity Due to Morphine or Hydromorphone Use in Renal Impairment: A Systematic Review. J Palliat Med SAGE Publications. 2016;19:1179–87. 10.1089/jpm.2016.0101. 10.1089/jpm.2016.0101 27399959

[85] Miller A, Price G. Gabapentin Toxicity in Renal Failure: The Importance of Dose Adjustment. Pain Med. 2009;10:190–2. 10.1111/j.1526-4637.2008.00492.x. 18721173 10.1111/j.1526-4637.2008.00492.x

[86] Brady M, Main J. Aciclovir neurotoxicity is an important side effect of therapy in patients with renal impairment. Clin Med Lond Engl. 2009;9:630. 10.7861/clinmedicine.9-6-630. 10.7861/clinmedicine.9-6-630 PMC4952315 20095318

[87] Liu M, Liang Y, Chigurupati S, Lathia JD, Pletnikov M, Sun Z, et al. Acute Kidney Injury Leads to Inflammation and Functional Changes in the Brain. J Am Soc Nephrol. 2008;19:1360–70. 10.1681/ASN.2007080901. 18385426 10.1681/ASN.2007080901 PMC2440297

[88] Ronco C, Haapio M, House AA, Anavekar N, Bellomo R, Cardiorenal Syndrome. J Am Coll Cardiol. 2008;52:1527–39. 10.1016/j.jacc.2008.07.051. 19007588 10.1016/j.jacc.2008.07.051

[89] Okpara R, Pena C, Nugent K. Cardiorenal Syndrome Type 3 Review. Cardiol Rev. 2024;32:140–5. 10.1097/CRD.0000000000000491. 36215106 10.1097/CRD.0000000000000491

[90] Pavan M. Incidence of Acute Cardiorenal Syndrome Type 3 in India. Iran J Kidney Dis. 2014;8:42–5. 24413720

[91] Drubel K, Marahrens B, Ritter O, Patschan D. Kidney-Related Outcome in Cardiorenal Syndrome Type 3. Int J Nephrol. 2022;2022:4895434. 10.1155/2022/4895434. 35178254 10.1155/2022/4895434 PMC8844349

[92] Faubel S. Acute kidney injury causes and exacerbates cardiac dysfunction. Am J Physiol-Ren Physiol Am Physiological Soc. 2023;324:F568–70. 10.1152/ajprenal.00305.2022. 10.1152/ajprenal.00305.2022 37167275

[93] Panwar R, McNicholas B, Teixeira JP, Kansal A. Renal perfusion pressure: role and implications in critical illness. Ann Intensive Care. 2025;15:115. 10.1186/s13613-025-01535-y. 40775567 10.1186/s13613-025-01535-y PMC12331573

[94] Legrand M, Dupuis C, Simon C, Gayat E, Mateo J, Lukaszewicz A-C, et al. Association between systemic hemodynamics and septic acute kidney injury in critically ill patients: a retrospective observational study. Crit Care. 2013;17:R278. 10.1186/cc13133. 24289206 10.1186/cc13133 PMC4056656

[95] Cosentino N, Marenzi G, Muratori M, Magrì D, Cattadori G, Agostoni P. Fluid balance in heart failure. Eur J Prev Cardiol. 2023;30:ii9–15. 10.1093/eurjpc/zwad166. 37819223 10.1093/eurjpc/zwad166

[96] Spano S, Maeda A, Lam J, Chaba A, Phongphithakchai A, Pattamin N, et al. A Pilot and Feasibility Study of Continuous Cardiac Output and Blood Pressure Monitoring during Intermittent Hemodialysis. Blood Purif [Internet]. 2024. 10.1159/000541201. [cited 2026 May 17]. 39222620 10.1159/000541201

[97] Rodríguez-Villar S, Kraut J, Arévalo-Serrano J, Sakka S, Harris C, Awad I, et al. Systemic acidemia impairs cardiac function in critically Ill patients. EClinicalMedicine. 2021;37:100956. 10.1016/j.eclinm.2021.100956. 34258569 10.1016/j.eclinm.2021.100956 PMC8255172

[98] Dépret F, Peacock WF, Liu KD, Rafique Z, Rossignol P, Legrand M. Management of hyperkalemia in the acutely ill patient. Ann Intensive Care. 2019;9:32. 10.1186/s13613-019-0509-8. 30820692 10.1186/s13613-019-0509-8 PMC6395464

[99] Di Lullo L, Reeves PB, Bellasi A, Ronco C. Cardiorenal Syndrome in Acute Kidney Injury. Semin Nephrol. 2019;39:31–40. 10.1016/j.semnephrol.2018.10.003. 30606406 10.1016/j.semnephrol.2018.10.003

[100] Lekawanvijit S. Cardiotoxicity of Uremic Toxins: A Driver of Cardiorenal Syndrome. Toxins. Multidisciplinary Digital Publishing Institute; 2018;10:352. 10.3390/toxins10090352 10.3390/toxins10090352 PMC6162485 30200452

[101] Kelly KJ. Distant Effects of Experimental Renal Ischemia/Reperfusion Injury. J Am Soc Nephrol. 2003;14:1549–58. 10.1097/01.ASN.0000064946.94590.46. 12761255 10.1097/01.asn.0000064946.94590.46

[102] Veltkamp DMJ, Zwank F, Groenestege WMT, Verhaar MC, van Solinge WW, Haitjema S, et al. Long-term risks of major adverse cardiovascular events after acute kidney injury: a systematic review and meta-analysis. Clin Kidney J. 2026;19:sfag145. 10.1093/ckj/sfag145. 42318101 10.1093/ckj/sfag145 PMC13273423

[103] Liaño F, Junco E, Pascual J, Madero R, Verde E. The spectrum of acute renal failure in the intensive care unit compared with that seen in other settings. The Madrid Acute Renal Failure Study Group. Kidney Int Suppl. 1998;66:S16–24. 9580541

[104] Bonavia A, Stiles N. Renohepatic crosstalk: a review of the effects of acute kidney injury on the liver. Nephrol Dial Transpl. 2022;37:1218–28. 10.1093/ndt/gfaa297. 10.1093/ndt/gfaa297 33527986

[105] Geavlete O, Ambrosy AP, Mebazaa A, Collins SP, Biegus J, Adamo M, et al. Liver Function Abnormalities in Acute Heart Failure: A State-of-the-art Review from an International Expert Group. Card Fail Rev. 2026;e17. 10.15420/cfr.2025.51. 10.15420/cfr.2025.51 PMC13392301 42495596

[106] Koch A, Horn A, Dückers H, Yagmur E, Sanson E, Bruensing J, et al. Increased liver stiffness denotes hepatic dysfunction and mortality risk in critically ill non-cirrhotic patients at a medical ICU. Crit Care. 2011;15:R266. 10.1186/cc10543. 22082207 10.1186/cc10543 PMC3388655

[107] Golab F, Kadkhodaee M, Zahmatkesh M, Hedayati M, Arab H, Schuster R, et al. Ischemic and non-ischemic acute kidney injury cause hepatic damage. Kidney Int. 2009;75:783–92. 10.1038/ki.2008.683. 19177157 10.1038/ki.2008.683

[108] Clementi A, Virzì GM, Sorbello M, Marzano N, Monciino P, Cabrera-Aguilar JS, et al. Hepato-Renal Crosstalk in Acute and Chronic Disease: From Shared Pathways to Therapeutic Targets. Biomedicines. 2025;13:1618. 10.3390/biomedicines13071618. 40722691 10.3390/biomedicines13071618 PMC12292868

[109] Dixon J, Lane K, MacPhee I, Philips B. Xenobiotic Metabolism: The Effect of Acute Kidney Injury on Non-Renal Drug Clearance and Hepatic Drug Metabolism. Int J Mol Sci. 2014;15:2538–53. 10.3390/ijms15022538. 24531139 10.3390/ijms15022538 PMC3958866

[110] Lane K, Dixon JJ, MacPhee IAM, Philips BJ. Renohepatic crosstalk: does acute kidney injury cause liver dysfunction? Nephrol Dial Transpl. 2013;28:1634–47. 10.1093/ndt/gft091. 10.1093/ndt/gft091 23685679

[111] Schädler D, Pausch C, Heise D, Meier-Hellmann A, Brederlau J, Weiler N, et al. The effect of a novel extracorporeal cytokine hemoadsorption device on IL-6 elimination in septic patients: A randomized controlled trial. PLOS ONE Public Libr Sci. 2017;12:e0187015. 10.1371/journal.pone.0187015. 10.1371/journal.pone.0187015 PMC5662220 29084247

[112] Supady A, Weber E, Rieder M, Lother A, Niklaus T, Zahn T, et al. Cytokine adsorption in patients with severe COVID-19 pneumonia requiring extracorporeal membrane oxygenation (CYCOV): a single centre, open-label, randomised, controlled trial. Lancet Respir Med. 2021;9:755–62. 10.1016/S2213-2600(21)00177-6. 34000236 10.1016/S2213-2600(21)00177-6 PMC8121541

[113] Heymann M, Schorer R, Putzu A. Mortality and adverse events of hemoadsorption with CytoSorb^®^ in critically ill patients: A systematic review and meta-analysis of randomized controlled trials. Acta Anaesthesiol Scand. 2022;66:1037–50. 10.1111/aas.14115. 35788557 10.1111/aas.14115 PMC9541789

[114] Foresto-Neto O, Menezes Silva L, Leite J, Silva M, Câmara N. Immunology of Kidney Disease. Annu Rev Immunol. 2024;42. 10.1146/annurev-immunol-090122-045843. 10.1146/annurev-immunol-090122-045843 38211945

[115] Kurts C, Von Vietinghoff S, Krebs CF, Panzer U. Kidney immunology from pathophysiology to clinical translation. Nat Rev Immunol. 2025;25:460–76. 10.1038/s41577-025-01131-y. 39885266 10.1038/s41577-025-01131-y

[116] Akcay A, Nguyen Q, Edelstein CL. Mediators of inflammation in acute kidney injury. Mediators Inflamm. 2009;2009:137072. 10.1155/2009/137072. 20182538 10.1155/2009/137072 PMC2825552

[117] White LE, Hassoun HT. Inflammatory Mechanisms of Organ Crosstalk during Ischemic Acute Kidney Injury. Int J Nephrol. 2012;2012:1–8. 10.4061/2012/505197. 10.4061/2012/505197 PMC3118535 21826270

[118] Singbartl K, Formeck CL, Kellum JA. Kidney-Immune System Crosstalk in AKI. Semin Nephrol. 2019;39:96–106. 10.1016/j.semnephrol.2018.10.007. 30606411 10.1016/j.semnephrol.2018.10.007

[119] Formeck CL, Feldman R, Althouse AD, Kellum JA. Risk and Timing of De Novo Sepsis in Critically Ill Children after Acute Kidney Injury. Kidney360. 2022;4:308–15. 10.34067/KID.0005082022. 10.34067/KID.0005082022 PMC10103342 36996298

[120] Lai T-S, Wang C-Y, Pan S-C, Huang T-M, Lin M-C, Lai C-F, et al. Risk of developing severe sepsis after acute kidney injury: a population-based cohort study. Crit Care. 2013;17:R231. 10.1186/cc13054. 24119576 10.1186/cc13054 PMC4056572

[121] Griffin BR, Teixeira JP, Ambruso S, Bronsert M, Pal JD, Cleveland JC, et al. Stage 1 acute kidney injury is independently associated with infection following cardiac surgery. J Thorac Cardiovasc Surg. 2021;161:1346–e13553. 10.1016/j.jtcvs.2019.11.004. 32007252 10.1016/j.jtcvs.2019.11.004 PMC7285820

[122] Bernier-Jean A, Beaubien-Souligny W, Ducruet T, Duca A, Albert M, Lavergne V, et al. Risk of de novo infection following acute kidney injury: A retrospective cohort study. J Crit Care. 2018;48:9–14. 10.1016/j.jcrc.2018.08.004. 30121515 10.1016/j.jcrc.2018.08.004

[123] Andres-Hernando A, Dursun B, Altmann C, Ahuja N, He Z, Bhargava R, et al. Cytokine production increases and cytokine clearance decreases in mice with bilateral nephrectomy. Nephrol Dial Transpl. 2012;27:4339–47. 10.1093/ndt/gfs256. 10.1093/ndt/gfs256 PMC3520082 22778179

[124] Bidar F, Peillon L, Bodinier M, Venet F, Monneret G, Lukaszewicz A-C, et al. Immune profiling of critically ill patients with acute kidney injury during the first week after various types of injuries: the REALAKI study. Crit Care. 2024;28:227. 10.1186/s13054-024-04998-w. 38978044 10.1186/s13054-024-04998-w PMC11232205

[125] Singbartl K, Miller L, Ruiz-Velasco V, Kellum JA. Reversal of Acute Kidney Injury–Induced Neutrophil Dysfunction: A Critical Role for Resistin*. Crit Care Med. 2016;44:e492–501. 10.1097/CCM.0000000000001472. 26646460 10.1097/CCM.0000000000001472 PMC4896858

[126] Wu H-P, Chuang L-P, Liu P-H, Chu C-M, Yu C-C, Lin S-W, et al. Decreased Monocyte HLA-DR Expression in Patients with Sepsis and Acute Kidney Injury. Med (Mex). 2022;58:1198. 10.3390/medicina58091198. 10.3390/medicina58091198 PMC9506340 36143874

[127] Selby NM, Kolhe NV, McIntyre CW, Monaghan J, Lawson N, Elliott D, et al. Defining the Cause of Death in Hospitalised Patients with Acute Kidney Injury. PLOS ONE Public Libr Sci. 2012;7:e48580. 10.1371/journal.pone.0048580. 10.1371/journal.pone.0048580 PMC3487783 23133643

[128] Bonavia A, Miller L, Kellum JA, Singbartl K. Hemoadsorption corrects hyperresistinemia and restores anti-bacterial neutrophil function. Intensive Care Med Exp. 2017;5:36. 10.1186/s40635-017-0150-5. 28779451 10.1186/s40635-017-0150-5 PMC5544662

[129] Zarbock A, Forni L, Koyner JL, Gómez H, Pannu N, Ostermann M, et al. Preventing acute kidney injury and its longer-term impact in the critically ill. Intensive Care Med. 2025;51:1331–47. 10.1007/s00134-025-08015-8. 40663138 10.1007/s00134-025-08015-8 PMC12283467

[130] Coca SG, Zabetian A, Ferket BS, Zhou J, Testani JM, Garg AX, et al. Evaluation of Short-Term Changes in Serum Creatinine Level as a Meaningful End Point in Randomized Clinical Trials. J Am Soc Nephrol. 2016;27:2529–42. 10.1681/ASN.2015060642. 26712525 10.1681/ASN.2015060642 PMC4978048

[131] Ikizler TA, Parikh CR, Himmelfarb J, Chinchilli VM, Liu KD, Coca SG, et al. A prospective cohort study of acute kidney injury and kidney outcomes, cardiovascular events, and death. Kidney Int. 2021;99:456–65. 10.1016/j.kint.2020.06.032. 32707221 10.1016/j.kint.2020.06.032 PMC7374148

[132] Horne KL, Viramontes-Hörner D, Packington R, Monaghan J, Shaw S, Akani A, et al. A comprehensive description of kidney disease progression after acute kidney injury from a prospective, parallel-group cohort study. Kidney Int. 2023;104:1185–93. 10.1016/j.kint.2023.08.005. 37611867 10.1016/j.kint.2023.08.005

[133] Zarbock A, Forni LG, Koyner JL, Bell S, Reis T, Meersch M, et al. Recommendations for clinical trial design in acute kidney injury from the 31st acute disease quality initiative consensus conference. A consensus statement. Intensive Care Med. 2024;50:1426–37. 10.1007/s00134-024-07560-y. 39115567 10.1007/s00134-024-07560-y PMC11377501

[134] Zoccali C, Lameire N, Ronco C, Carlson N, Faguer S, Selby N, et al. Acute kidney injury, acute kidney disease and chronic kidney disease: challenges and research perspectives. Nephrol Dial Transpl. 2025;40:1650–8. 10.1093/ndt/gfaf090. 10.1093/ndt/gfaf090 40353771

[135] Siew ED, Matheny ME, Ikizler TA, Lewis JB, Miller RA, Waitman LR, et al. Commonly used surrogates for baseline renal function affect the classification and prognosis of acute kidney injury. Kidney Int. 2010;77:536–42. 10.1038/ki.2009.479. 20042998 10.1038/ki.2009.479 PMC2929703

[136] Thongprayoon C, Cheungpasitporn W, Harrison AM, Kittanamongkolchai W, Ungprasert P, Srivali N, et al. The comparison of the commonly used surrogates for baseline renal function in acute kidney injury diagnosis and staging. BMC Nephrol. 2016;17:6. 10.1186/s12882-016-0220-z. 26748909 10.1186/s12882-016-0220-z PMC4707008

[137] Hsu C, Chinchilli VM, Coca S, Devarajan P, Ghahramani N, Go AS, et al. Post–Acute Kidney Injury Proteinuria and Subsequent Kidney Disease Progression: The Assessment, Serial Evaluation, and Subsequent Sequelae in Acute Kidney Injury (ASSESS-AKI) Study. JAMA Intern Med. 2020;180:402. 10.1001/jamainternmed.2019.6390. 31985750 10.1001/jamainternmed.2019.6390 PMC6990681

[138] Murphy DP, Wolfson J, Reule S, Johansen KL, Ishani A, Drawz PE. Renin–Angiotensin–Aldosterone System Blockade after AKI with or without Recovery among US Veterans with Diabetic Kidney Disease. J Am Soc Nephrol. 2023;34:1721–32. 10.1681/ASN.0000000000000196. 37545022 10.1681/ASN.0000000000000196 PMC10561814

[139] Chen J-Y, Tsai I-J, Pan H-C, Liao H-W, Neyra JA, Wu V-C, et al. The Impact of Angiotensin-Converting Enzyme Inhibitors or Angiotensin II Receptor Blockers on Clinical Outcomes of Acute Kidney Disease Patients: A Systematic Review and Meta-Analysis. Front Pharmacol. 2021;12:665250. 10.3389/fphar.2021.665250. 34354583 10.3389/fphar.2021.665250 PMC8329451

[140] Tan BWL, Tan BWQ, Akalya K, Hong W-Z, Da Y, Low S, et al. Effect of Post-Acute Kidney Injury Use of Renin-Angiotensin Inhibitors on Long-term Mortality and Major Adverse Kidney Events: A 5-year Retrospective Observational Cohort Study. Kidney Med. 2025;7:100996. 10.1016/j.xkme.2025.100996. 40321973 10.1016/j.xkme.2025.100996 PMC12049942

[141] Pan H-C, Chen J-Y, Chen H-Y, Yeh F-Y, Huang TT-M, Sun C-Y, et al. Sodium-Glucose Cotransport Protein 2 Inhibitors in Patients With Type 2 Diabetes and Acute Kidney Disease. JAMA Netw Open. 2024;7:e2350050. 10.1001/jamanetworkopen.2023.50050. 38170522 10.1001/jamanetworkopen.2023.50050 PMC10765268

[142] Pan H-C, Chen J-Y, Chen H-Y, Yeh F-Y, Sun C-Y, Huang TT-M, et al. GLP-1 receptor agonists’ impact on cardio-renal outcomes and mortality in T2D with acute kidney disease. Nat Commun. 2024;15:5912. 10.1038/s41467-024-50199-y. 39003287 10.1038/s41467-024-50199-y PMC11246471

[143] Palmowski L, Lindau S, Henk LC, Marko B, Witowski A, Nowak H, et al. Predictive enrichment for the need of renal replacement in sepsis-associated acute kidney injury: combination of furosemide stress test and urinary biomarkers TIMP-2 and IGFBP-7. Ann Intensive Care. 2024;14:111. 10.1186/s13613-024-01349-4. 39002065 10.1186/s13613-024-01349-4 PMC11246358

[144] Weidhase L, Wille S, Foede H, Gilch F, Mende M, Scharf-Janßen C, et al. Spontaneous diuresis in combination with furosemide stress test (SD-FST) as predictor for successful liberation from kidney replacement therapy: a prospective observational study. Crit Care. 2025;29:214. 10.1186/s13054-025-05452-1. 40420285 10.1186/s13054-025-05452-1 PMC12107999

[145] Vasquez-Rios G, Moledina DG, Jia Y, McArthur E, Mansour SG, Thiessen-Philbrook H, et al. Pre-operative kidney biomarkers and risks for death, cardiovascular and chronic kidney disease events after cardiac surgery: the TRIBE-AKI study. J Cardiothorac Surg. 2022;17:338. 10.1186/s13019-022-02066-4. 36567329 10.1186/s13019-022-02066-4 PMC9790121

[146] Wilson M, Packington R, Sewell H, Bartle R, McCole E, Kurth MJ, et al. Biomarkers During Recovery From AKI and Prediction of Long-term Reductions in Estimated GFR. Am J Kidney Dis. 2022;79:646–e6561. 10.1053/j.ajkd.2021.08.017. 34653541 10.1053/j.ajkd.2021.08.017

[147] Coca SG, Vasquez-Rios G, Mansour SG, Moledina DG, Thiessen-Philbrook H, Wurfel MM, et al. Plasma Soluble Tumor Necrosis Factor Receptor Concentrations and Clinical Events After Hospitalization: Findings From the ASSESS-AKI and ARID Studies. Am J Kidney Dis. 2023;81:190–200. 10.1053/j.ajkd.2022.08.007. 36108888 10.1053/j.ajkd.2022.08.007 PMC9868060

[148] Moon SJ, Park HB, Yoon SY, Lee SC. Urinary Biomarkers for Early Detection of Recovery in Patients with Acute Kidney Injury. J Korean Med Sci. 2013;28:1181. 10.3346/jkms.2013.28.8.1181. 23960445 10.3346/jkms.2013.28.8.1181 PMC3744706

[149] Meersch M, Schmidt C, Van Aken H, Martens S, Rossaint J, Singbartl K et al. Urinary TIMP-2 and IGFBP7 as Early Biomarkers of Acute Kidney Injury and Renal Recovery following Cardiac Surgery. Rosenberger P, editor. PLoS ONE. 2014;9:e93460. 10.1371/journal.pone.0093460 10.1371/journal.pone.0093460 PMC3968141 24675717

[150] Göcze I, Jauch D, Götz M, Kennedy P, Jung B, Zeman F, et al. Biomarker-guided Intervention to Prevent Acute Kidney Injury After Major Surgery: The Prospective Randomized BigpAK Study. Ann Surg. 2018;267:1013–20. 10.1097/SLA.0000000000002485. 28857811 10.1097/SLA.0000000000002485

[151] Zarbock A, Ostermann M, Forni L, Bode C, Wild L, Putensen C, et al. A preventive care strategy to reduce moderate or severe acute kidney injury after major surgery (BigpAK-2); a multinational, randomised clinical trial. Lancet Elsevier. 2025;406:2782–91. 10.1016/S0140-6736(25)01717-9. 10.1016/S0140-6736(25)01717-9 41242333

[152] Meersch M, Schmidt C, Hoffmeier A, Van Aken H, Wempe C, Gerss J, et al. Prevention of cardiac surgery-associated AKI by implementing the KDIGO guidelines in high risk patients identified by biomarkers: the PrevAKI randomized controlled trial. Intensive Care Med. 2017;43:1551–61. 10.1007/s00134-016-4670-3. 28110412 10.1007/s00134-016-4670-3 PMC5633630

[153] Srisawat N, Laoveeravat P, Limphunudom P, Lumlertgul N, Peerapornratana S, Tiranathanagul K, et al. The effect of early renal replacement therapy guided by plasma neutrophil gelatinase associated lipocalin on outcome of acute kidney injury: A feasibility study. J Crit Care. 2018;43:36–41. 10.1016/j.jcrc.2017.08.029. 28843662 10.1016/j.jcrc.2017.08.029

[154] Wald R, Adhikari NKJ, Smith OM, Weir MA, Pope K, Cohen A, et al. Comparison of standard and accelerated initiation of renal replacement therapy in acute kidney injury. Kidney Int. 2015;88:897–904. 10.1038/ki.2015.184. 26154928 10.1038/ki.2015.184

[155] Liu KD, Siew ED, Tuot DS, Vijayan A, Matzumura Umemoto G, Birkelo BC, et al. A Conservative Dialysis Strategy and Kidney Function Recovery in Dialysis-Requiring Acute Kidney Injury: The Liberation From Acute Dialysis (LIBERATE-D) Randomized Clinical Trial. JAMA. 2026;335:326. 10.1001/jama.2025.21530. 41201895 10.1001/jama.2025.21530 PMC12595548

[156] Vijayan A, Delos Santos RB, Li T, Goss CW, Palevsky PM. Effect of Frequent Dialysis on Renal Recovery: Results From the Acute Renal Failure Trial Network Study. Kidney Int Rep. 2018;3:456–63. 10.1016/j.ekir.2017.11.018. 29725650 10.1016/j.ekir.2017.11.018 PMC5932307

[157] McCoy IE, Liu KD, Ghamarian E, Quenot J-P, Zarbock A, Bihorac A, et al. Dialysis Dependence in Standard versus Accelerated Initiation of KRT in AKI: A Post Hoc Analysis. Clin J Am Soc Nephrol. 2025;20:601–7. 10.2215/CJN.0000000672. 40232884 10.2215/CJN.0000000672 PMC12097186

[158] Legrand M, Bagshaw SM, Bhatraju PK, Bihorac A, Caniglia E, Khanna AK, et al. Sepsis-associated acute kidney injury: recent advances in enrichment strategies, sub-phenotyping and clinical trials. Crit Care. 2024;28:92. 10.1186/s13054-024-04877-4. 38515121 10.1186/s13054-024-04877-4 PMC10958912

[159] The STARRT-AKI, Investigators. Timing of Initiation of Renal-Replacement Therapy in Acute Kidney Injury. N Engl J Med. 2020;383:240–51. 10.1056/NEJMoa2000741. 32668114 10.1056/NEJMoa2000741

[160] Parapiboon W, Tatiyanupanwong S, Khositrangsikun K, Phulkerd T, Kaewdoungtien P, Pichitporn W, et al. Lower-Dosage Acute Peritoneal Dialysis versus Acute Intermittent Hemodialysis in Acute Kidney Injury: A Randomized Controlled Trial. Clin J Am Soc Nephrol. 2024;19:970–7. 10.2215/CJN.0000000000000482. 38809614 10.2215/CJN.0000000000000482 PMC11321733

[161] Gaudry S, Hajage D, Martin-Lefevre L, Lebbah S, Louis G, Moschietto S, et al. Comparison of two delayed strategies for renal replacement therapy initiation for severe acute kidney injury (AKIKI 2): a multicentre, open-label, randomised, controlled trial. Lancet. 2021;397:1293–300. 10.1016/S0140-6736(21)00350-0. 33812488 10.1016/S0140-6736(21)00350-0

[162] Barbar SD, Clere-Jehl R, Bourredjem A, Hernu R, Montini F, Bruyère R, et al. Timing of Renal-Replacement Therapy in Patients with Acute Kidney Injury and Sepsis. N Engl J Med. 2018;379:1431–42. 10.1056/NEJMoa1803213. 30304656 10.1056/NEJMoa1803213

[163] Zampieri FG, Serpa-Neto A, Wald R, Bellomo R, Bagshaw SM. Hierarchical endpoints in critical care: A post-hoc exploratory analysis of the standard versus accelerated initiation of renal-replacement therapy in acute kidney injury and the intensity of continuous renal-replacement therapy in critically ill patients trials. J Crit Care. 2024;82:154767. 10.1016/j.jcrc.2024.154767. 38461657 10.1016/j.jcrc.2024.154767

[164] Cerdá J, Liu KD, Cruz DN, Jaber BL, Koyner JL, Heung M, et al. Promoting Kidney Function Recovery in Patients with AKI Requiring RRT. Clin J Am Soc Nephrol. 2015;10:1859–67. 10.2215/CJN.01170215. 26138260 10.2215/CJN.01170215 PMC4594060

[165] Singh G, Hu Y, Jacobs S, Brown J, George J, Bermudez M, et al. Post-Discharge Mortality and Rehospitalization among Participants in a Comprehensive Acute Kidney Injury Rehabilitation Program. Kidney360. 2021;2:1424–33. 10.34067/KID.0003672021. 35373103 10.34067/KID.0003672021 PMC8786140

[166] Schulman IH, Chan K, Der JS, Wilkins KJ, Corns HL, Sayer B, et al. Readmission and Mortality After Hospitalization With Acute Kidney Injury. Am J Kidney Dis. 2023;82:63–e741. 10.1053/j.ajkd.2022.12.008. 37115159 10.1053/j.ajkd.2022.12.008 PMC10293057

[167] May HP, Herges JR, Anderson BK, Hanson GJ, Kashani KB, Kattah AG, et al. Posthospital Multidisciplinary Care for AKI Survivors: A Feasibility Pilot. Kidney Med. 2023;5:100734. 10.1016/j.xkme.2023.100734. 37964784 10.1016/j.xkme.2023.100734 PMC10641567

[168] Mayer KP, Ortiz-Soriano VM, Kalantar A, Lambert J, Morris PE, Neyra JA. Acute kidney injury contributes to worse physical and quality of life outcomes in survivors of critical illness. BMC Nephrol. 2022;23:137. 10.1186/s12882-022-02749-z. 35392844 10.1186/s12882-022-02749-z PMC8991933

[169] Chaïbi K, Ehooman F, Pons B, Martin-Lefevre L, Boulet E, Boyer A, et al. Long-term outcomes after severe acute kidney injury in critically ill patients: the SALTO study. Ann Intensive Care. 2023;13:18. 10.1186/s13613-023-01108-x. 36907976 10.1186/s13613-023-01108-x PMC10008759

[170] Huang F, Ethier I, Vaillant I, Silver SA, Côté J-M. Self-Reported Burden and Health-Related Quality of Life in Acute Kidney Injury Survivors Compared with Patients with Advanced CKD. Kidney360. 2025;6:720–7. 10.34067/KID.0000000707. 40440456 10.34067/KID.0000000707 PMC12136637

[171] Toonstra AL, Zanni JM, Sperati CJ, Nelliot A, Mantheiy E, Skinner EH, et al. Feasibility and Safety of Physical Therapy during Continuous Renal Replacement Therapy in the Intensive Care Unit. Ann Am Thorac Soc. 2016;13:699–704. 10.1513/AnnalsATS.201506-359OC. 26788890 10.1513/AnnalsATS.201506-359OC

[172] Mayer KP, Joseph-Isang E, Robinson LE, Parry SM, Morris PE, Neyra JA. Safety and Feasibility of Physical Rehabilitation and Active Mobilization in Patients Requiring Continuous Renal Replacement Therapy: A Systematic Review. Crit Care Med. 2020;48:e1112–20. 10.1097/CCM.0000000000004526. 33001619 10.1097/CCM.0000000000004526

[173] Mayer KP, Hornsby AR, Soriano VO, Lin TC, Cunningham JT, Yuan H, et al. Safety, Feasibility, and Efficacy of Early Rehabilitation in Patients Requiring Continuous Renal Replacement: A Quality Improvement Study. Kidney Int Rep. 2020;5:39–47. 10.1016/j.ekir.2019.10.003. 31922059 10.1016/j.ekir.2019.10.003 PMC6943757

[174] Jung B, Jabaudon M, De Jong A, Bitker L, Audard J, Klouche K, et al. Sodium Bicarbonate for Severe Metabolic Acidemia and Acute Kidney Injury: The BICARICU-2 Randomized Clinical Trial. JAMA. 2025;334:2000. 10.1001/jama.2025.20231. 41159812 10.1001/jama.2025.20231 PMC12573113

[175] Serpa Neto A, Naorungroj T, Gallagher M, Bellomo R. Impact of Intensity of Continuous Renal Replacement Therapy on Duration of Ventilation in Critically Ill Patients: A Secondary Analysis of the RENAL Trial. Blood Purif. 2023;52:888–97. 10.1159/000533687. 37852200 10.1159/000533687

[176] Demirjian S, Teo BW, Guzman JA, Heyka RJ, Paganini EP, Fissell WH, et al. Hypophosphatemia during continuous hemodialysis is associated with prolonged respiratory failure in patients with acute kidney injury. Nephrol Dial Transpl. 2011;26:3508–14. 10.1093/ndt/gfr075. 10.1093/ndt/gfr075 21382993

[177] Neyra JA, Thompson Bastin M, Ghamarian E, Firenzuoli F, Takeuchi T, Bagshaw SM, et al. Hypophosphatemia During Kidney Replacement Therapy and Ventilator-Free Days: A Post Hoc Analysis of the STARRT-AKI Trial. Am J Kidney Dis. 2026;87:654–e6641. 10.1053/j.ajkd.2025.11.012. 41763519 10.1053/j.ajkd.2025.11.012 PMC13420734

[178] Kelly YP, Da Costa BR, Beaubien-Souligny W, Clark EG, Murray PT, Nichol A, et al. Factors associated with adverse haemodynamic events during the STARRT-AKI trial: a post-hoc secondary analysis. Crit Care. 2025;29:534. 10.1186/s13054-025-05693-0. 41466322 10.1186/s13054-025-05693-0 PMC12751851

[179] Quickfall D, La A, Pisano J, Costello P, Gunning S, Koyner JL. Infections and mortality in ICU patients undergoing continuous renal replacement therapy: a retrospective cohort study. BMC Nephrol. 2025;26:321. 10.1186/s12882-025-04272-3. 40596911 10.1186/s12882-025-04272-3 PMC12220524

[180] Zakkar M, Bruno VD, Guida G, Angelini GD, Chivasso P, Suleiman MS, et al. Postoperative acute kidney injury defined by RIFLE criteria predicts early health outcome and long-term survival in patients undergoing redo coronary artery bypass graft surgery. J Thorac Cardiovasc Surg. 2016;152:235–42. 10.1016/j.jtcvs.2016.02.047. 27016793 10.1016/j.jtcvs.2016.02.047 PMC4915911

[181] Eslami P, Hekmat M, Beheshti M, Baghaei R, Mirhosseini SM, Pourmotahari F, et al. A Randomized, Double-Blind, Placebo-Controlled, Clinical Trial of High-Dose, Short-Term Vitamin D Administration in the Prevention of Acute Kidney Injury after Cardiac Surgery. Cardiorenal Med. 2021;11:52–8. 10.1159/000511058. 33498049 10.1159/000511058

